# Supplementary material for: Outcomes for university students following emergency care presentation for deliberate self-harm: a retrospective observational study of emergency departments in England for 2017/2018
Source: BMJ Open. 2024 Feb 6;14(2):e078672. doi: 10.1136/bmjopen-2023-078672 (PMC10860022; doi:10.1136/bmjopen-2023-078672)
Supplement: Supplementary data [file bmjopen-2023-078672supp001.pdf]

Supplementary material to the paper titled "Outcomes for university students following emergency care presentation for deliberate self-harm: retrospective observational study of Emergency Departments in England for 2017/2018"

Authors: Catherine Campbell, Joe Dodd, Igor Francetic

Version: January 2024.

Appendix 1: Distribution of LSOAs with more than 10 percent of residents aged 18-23

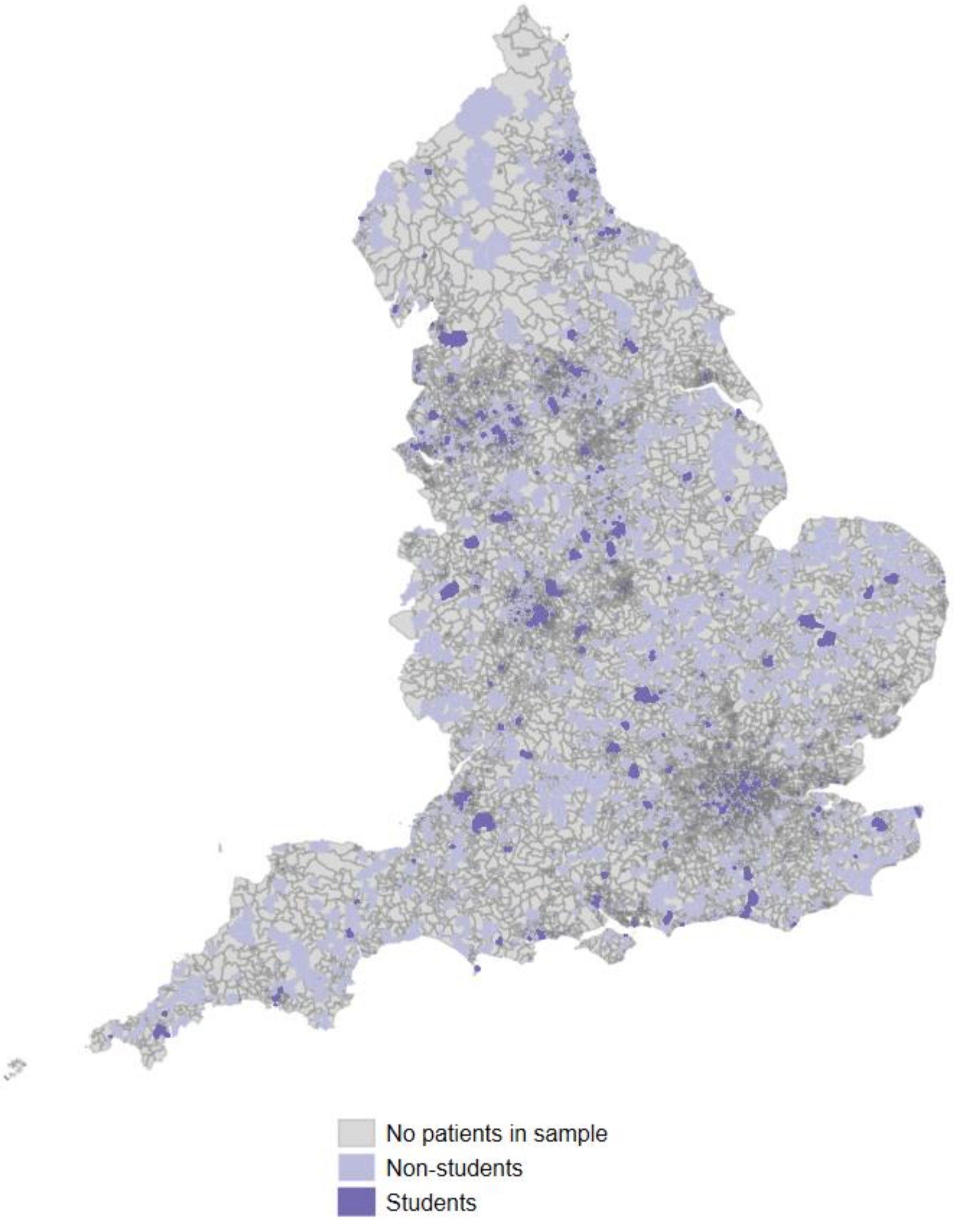

Note: Grey areas represent LSOAs in England that are not represented in our sample. Areas in light purple represent LSOAs of residence for patients not identified as students in our sample. Areas in darker purple represent LSOAs of residence of patients identified as students. The latter are clustered around university areas in England, for example Exeter, Bristol, Bath, Reading, London, Oxford, Cambridge, Birmingham, Coventry, Norwich, Liverpool, Manchester, Leeds, Sheffield, York, Lancaster, Durham and Newcastle among others.

Appendix 2: Distribution of primary ED codes (severity proxy) for analytical sample of attendances

Primary ED Diagnosis	Freq.	Percent
Laceration	1,244	8.84
Contusion/abrasion	360	2.56
Soft tissue inflammation	95	0.68
Head injury	108	0.77
Dislocation/fracture/joints	281	2.00
Sprain/ligament injury	107	0.76
Muscle/tendon injury	43	0.31
Burns and scalds	33	0.23
Electric shock	1	0.01
Foreign body	227	1.61
Bites/stings	13	0.09
Poisoning (inc overdose)	4,780	33.96
Near drowning	2	0.01
Visceral injury	6	0.04
Infectious disease	3	0.02
Local infection	24	0.17
Septicaemia	1	0.01
Cardiac conditions	28	0.20
Cerebro-vascular conditions	3	0.02
Other vascular conditions	2	0.01
Haematological conditions	4	0.03
Central nervous system conditions	43	0.31
Respiratory conditions	14	0.10
Gastrointestinal conditions	60	0.43
Urological conditions (inc cystitis)	12	0.09
Obstetric conditions	1	0.01
Gynaecological conditions	36	0.26
Diabetes and other endocrinological conditions	15	0.11
Dermatological conditions	6	0.04
Allergy (inc anaphylaxis)	3	0.02
Facio-maxillary conditions	2	0.01
ENT conditions	8	0.06
Psychiatric conditions	2,154	15.30
Ophthalmological conditions	17	0.12
Social problems (inc chronic alcoholism)	94	0.67
Diagnosis not classifiable	1,407	10.00
Nothing abnormal detected	189	1.34
Missclassified	703	5.00
No/missing diagnosis	1,945	13.82
Total	14,074	100
