# Supplementary material for: Outcomes for university students following emergency care presentation for deliberate self-harm: a retrospective observational study of emergency departments in England for 2017/2018
Source: BMJ Open. 2024 Feb 6;14(2):e078672. doi: 10.1136/bmjopen-2023-078672 (PMC10860022; doi:10.1136/bmjopen-2023-078672)
Supplement: Author's manuscript [file bmjopen-2023-078672.draft_revisions.pdf]

BMJ Open is committed to open peer review. As part of this commitment we make the peer review history of every article we publish publicly available.

When an article is published we post the peer reviewers' comments and the authors' responses online. We also post the versions of the paper that were used during peer review. These are the versions that the peer review comments apply to.

The versions of the paper that follow are the versions that were submitted during the peer review process. They are not the versions of record or the final published versions. They should not be cited or distributed as the published version of this manuscript.

BMJ Open is an open access journal and the full, final, typeset and author-corrected version of record of the manuscript is available on our site with no access controls, subscription charges or pay-per-view fees (http://bmjopen.bmj.com).

If you have any questions on BMJ Open's open peer review process please email info.bmjopen@bmj.com

BMJ Open

Outcomes for university students following emergency care presentation for deliberate self-harm: retrospective observational study of Emergency Departments in England for 2017/2018

Journal:	*BMJ Open*
Manuscript ID	bmjopen-2023-078672
Article Type:	Original research
Date Submitted by the Author:	08-Aug-2023
Complete List of Authors:	Campbell, Catherine; The University of Manchester, Centre for Primary Care and Health Services Research<br>Dodd, Joe; The University of Manchester, Centre for Primary Care and Health Services Research<br>Francetic, Igor; The University of Manchester, Centre for Primary Care and Health Services Research
Keywords:	Suicide & self-harm < PSYCHIATRY, ACCIDENT & EMERGENCY MEDICINE, Patients, MENTAL HEALTH

SCHOLARONE™
Manuscripts

I, the Submitting Author has the right to grant and does grant on behalf of all authors of the Work (as defined in the below author licence), an exclusive licence and/or a non-exclusive licence for contributions from authors who are: i) UK Crown employees; ii) where BMJ has agreed a CC-BY licence shall apply, and/or iii) in accordance with the terms applicable for US Federal Government officers or employees acting as part of their official duties; on a worldwide, perpetual, irrevocable, royalty-free basis to BMJ Publishing Group Ltd ("BMJ") its licensees and where the relevant Journal is co-owned by BMJ to the co-owners of the Journal, to publish the Work in this journal and any other BMJ products and to exploit all rights, as set out in our licence.

The Submitting Author accepts and understands that any supply made under these terms is made by BMJ to the Submitting Author unless you are acting as an employee on behalf of your employer or a postgraduate student of an affiliated institution which is paying any applicable article publishing charge ("APC") for Open Access articles. Where the Submitting Author wishes to make the Work available on an Open Access basis (and intends to pay the relevant APC), the terms of reuse of such Open Access shall be governed by a Creative Commons licence – details of these licences and which Creative Commons licence will apply to this Work are set out in our licence referred to above.

Other than as permitted in any relevant BMJ Author's Self Archiving Policies, I confirm this Work has not been accepted for publication elsewhere, is not being considered for publication elsewhere and does not duplicate material already published. I confirm all authors consent to publication of this Work and authorise the granting of this licence.

For peer review only

Title: Outcomes for university students following emergency care presentation for deliberate self-harm: retrospective observational study of Emergency Departments in England for 2017/2018

Authors: Catherine Campbell, Research Assistant [a], Joe Dodd [a], Research Associate, Igor Francetic, Research Fellow[a]✉

Affiliation: [a] Health Organisation, Policy and Economics (HOPE) group, School of Health Sciences, University of Manchester, UK.

✉ **Corresponding author**: Igor Francetic, Health Organisation, Policy and Economics (HOPE) group, School of Health Sciences, University of Manchester, Williamson Building, Oxford Road, M13 9PL, Manchester, UK. Email: igor.francetic@manchester.ac.uk.

Abstract

- **Objectives:** Identify university-aged students and contrast their healthcare provision and outcomes with other patient groups attending emergency departments for deliberate self-harm.
- **Design:** Retrospective cross-sectional observational study.
- **Setting:** Patients visiting 129 public hospital Emergency Departments across England between April 2017 and March 2018.
- **Participants:** 62,405 patients visiting Emergency Departments for conditions linked to deliberate self-harm, 2,952 of which were identified as university-aged students.
- **Primary and secondary outcome measures:** We study various outcomes across the entire patient pathway in the Emergency Department: Waiting time to initial assessment upon arrival at the emergency department, count of investigations delivered, discharge destination (patients refusing treatment or leave before being seen, referred to another provider or admitted to inpatient care, discharged with no follow-up) and unplanned follow-up visit within 7 days.
- **Results:** We find a statistically significant difference of 0.232 [-0.360,-0.104] less investigations delivered to students compared to non-students (about 7 percent compared to the baseline number of investigations). Stratified analyses reveal that students visiting the Emergency Department during regular daily hours (6AM-6PM) are 1.8 [0.31, 3.29] percent more likely to leave before treatment or refuse treatment, and receive 0.372 [-0.567,-0.178] less investigations. Reattendance within 7 days is lower among students visiting Emergency Departments during out of hours (-2.21 [-3.84,-0.58] percent) or arriving by ambulance (-1.98 [-3.41,-0.55] percent) compared to non-students.
- **Conclusions**: Whist there is weak evidence of less intense investigations being delivered, there is an increased likelihood of walk-outs during regular daytime hours among students compared to other patients visiting Emergency Departments after an episode of deliberate self-harm. Given the high risk of suicide attempts after episodes of deliberate self-harm among students, our findings may highlight the need for more focused interventions on this group of patients.

For peer review only

Version: August 23

Word count: 4,271

Strengths and limitations of this study

- We propose an approach to identify students in administrative records for Emergency Departments in England, focusing on the population of attendances between April 2017 and March 2018
- Supply-side confounders are accounted by a high-dimensional fixed effect, accounting for Emergency Department specific combinations of day of the week and month of the year
- We study outcomes across the entire pathway for patients attending Emergency Departments after episodes of deliberate self-harm, comparing students and non-students

For peer review only

Title: Outcomes for university students following emergency care presentation for deliberate self-harm: retrospective observational study

Introduction

Attending university can be stressful in regard to coping with academic pressure, managing work responsibilities and living far from social networks developed during early adolescence. This stressful environment poses a problem to the increasing number of young adults with mental health conditions entering university [1]. In the United States this is particularly observable with 88% of counselling centre's directors reporting an increase in severe psychological problems over the previous five years, among 274 surveyed universities [2]. Moreover, the age at which students attend university may have a confounding effect on the stressful environment of university as 75% of individuals with a mental health disorder encounter their first episode by the age of 25 [3]. As a result, increased research on student mental health is now being requested [4]. In England, trends in incidence of severe mental health problems and suicide do not seem to be higher among students compared to the general population of the same age [5–7]. Nevertheless, from a service provision perspective, the student population appears particularly vulnerable. For example, evidence suggests availability and accessibility of mental health services for students is often limited [8–10].

Our study relates to literature surrounding the of quality of care within emergency departments (EDs) for deliberate self-harm patients. To date, there are several studies investigating the impact of staff attitudes and perception of mental health on quality of care outcomes for this group. Firstly, ED staff attitude can exert a crucial effect on the future health-seeking behaviours of self-harm patients [11]. This suggests if a patient experiences a negative reaction to seeking help for their ailment, they are less likely to seek help again in the same way. Moreover, patients who self-harm or attempt suicide are found to be more negatively evaluated and viewed as "wasting resources" compared with patients attending for other reasons [12]. Finally, Timson et al. [13] find a correlation between staff knowledge of mental health treatment and negativity towards self-harm patients. These studies highlight the potential issues a student attending ED for deliberate self-harm may face.

For peer review only

There is a limited body of literature on the provision of care to students during ED attendances. This paper speaks to the high attendance volumes at emergency departments for deliberate self-harm (DSH) by university students. Holt and Powell [14] find that 80 percent of university student attendees are not registered with a GP, reflecting an apparent misuse of emergency care services. This has been attributed to students failing to seek healthcare on university campuses' due to a fear of vulnerability and breach of confidentiality [15]. Importantly, treatment during emergency attendances for self-harm is particularly sensitive because there is ample evidence relating hospital attendance for non-fatal self-harm to subsequent risk of completed and attempted suicide [16–19]. Coupled with the relatively high proportion of university students attending emergency departments for deliberate self-harm compared with attendances for other reasons, this underscores the importance of understanding the type of treatment that these patients receive.

Building on these stylised facts, we aim to explicitly study the healthcare provision and outcomes of university-aged students attending emergency departments for deliberate self-harm, contrasting them with other DSH patients. In doing so, given that administrative hospital records do not include information about student status, we propose a proxy indicator to identify students in Hospital Episode Statistics (HES) records.

Methods

Data and sample

Our study builds on the attendance records of patients visiting Emergency Departments across England between April 2017 and March 2018. These records were obtained from the Hospital Episode Statistics (HES). HES contains routinely collected of data covering procedures and treatments for all patients visiting secondary care facilities across England, tracking the patients' pathway from attendance to discharge. Attendance records contain an extensive range of information about patients, including the hospital site, admission and discharge dates and times, GP practice to which the patient is registered, and a comprehensive set of demographic information. The HES also captures the patient's small geographical area of residence. This is at the Lower layer Super Output Area (LSOAs) level, which includes

For peer review only

approximately 1,500 individuals. These are used to link information on area-level of deprivation, namely the income component of the Index of Multiple Deprivation [20].

From the Office for National Statistics, we obtain data on the demographic structure of LSOAs. We use this to identify areas with a disproportionately higher share of individuals aged 18-25, an age-range consistent with being a university student. From regular NHS Digital public releases [21] we obtained the number of patients registered with GP practices divided in 5-year age groups. For each GP practice we computed the share of young patients aged between 14 and 44.

Our target population is represented by patients aged 10 or older visiting EDs in England between April 2017 and March 2018, whose attendance was related to an episode of DSH (n=97,047). Any observation without a recorded LSOA is excluded. We focus on this period because of limitations in access to more recent data. Incidentally, this protects our analysis from any disruptions to services caused by the COVID-19 pandemic. We exclude children under 10 as their patterns and motivations for self-harm are likely to be fundamentally different from those of adolescent and adult patients [22].

To obtain our analytical sample, we exclude attendances with missing or implausible values on key variables, and patients who were dead upon arrival at the ED (n=31,338). To avoid ambiguity, we also exclude the few patients appearing more than once on the same day in the dataset (n=514). Furthermore, we restrict our analysis to patients attending Type 1 EDs; that is 24-hour consultant-led services with full facilities for resuscitating patients. We do this as Type 2 and Type 3 EDs tend to have a different and more heterogeneous mix of patients [23]. Our final analytical sample is composed by 62,405 attendances categorised as related to episodes of deliberate self-harm, across 127 EDs in England, between April 2017 and March 2018. In Table 1 below we show descriptive statistics of our main covariates for the entire sample and for the two subgroups defined by our exposure.

For peer review only

Exposure: Proxy indicator for university students visiting EDs in England

Our main exposure is represented by a proxy indicator which aims to identify university students visiting an ED in England based on HES Accident and Emergency (A&E) records. This proxy attempts to circumvent the absence of a specific variable indicating whether a patient is a student. Firstly, we define 18 to 23 as an age-range consistent with most university students. Secondly, we identify LSOAs where more than 10 percent of residents are aged between 18 and 23. This second criterion tries to identify areas in England where student residences are located. Thirdly, we identify GP practices whose patients are predominantly within an age range consistent with university students [24]. Specifically, we flagged 5,527 DSH attendance from patients registered with GP practices with over 50% of patients aged 14 to 44, hence likely to treat students. Taken together, we identify patients aged 18 to 23 who either live in one of the LSOAs likely to include student accommodation or are registered with a GP practice likely to serve students. In our sample, 2,952 ED attendances (4.73 percent of the sample) met these criteria.

Outcomes

Our analysis aims to study the healthcare provision and outcomes of university-aged students attending emergency departments for deliberate self-harm, contrasting them with other DSH patients. The outcomes studied cover the entire patient pathway in ED from arrival to discharge. However, we do not focus on the choice of specific treatments delivered and their intensity as these are likely to depend on patient severity, which we are unable to measure. We firstly look at the time to initial assessment upon arrival at the ED. Secondly, we look at the count of investigations delivered. Thirdly, we look at various discharge destinations: patients refusing treatment or leave before being seen, referred to another provider or admitted to inpatient care, discharged with no follow-up. Finally, we measure unplanned follow-up visit within 7 days.

Empirical approach

A simple comparison in means across groups of patients by student status is unlikely to give an accurate account of differences in outcomes due to various sources of confounding. For example, patients flagged

For peer review only - http://bmjopen.bmj.com/site/about/guidelines.xhtml

as students may have more or less severe conditions than other DSH patients. They may also systematically visit EDs that are intrinsically different or at times where EDs are more or less busy or staffed.

To study whether being a student is associated with differences in outcomes and treatments compared to other DHS patients visiting an English ED, we conduct a regression-based analysis.

Our main empirical specification is:

$$y_{ihmd} = \beta \times \text{Student}_i + \gamma \times \text{Ind}_i + \omega \times \text{Att}_{imd} + \delta\ \text{Severity}_{ihmd} + \text{HDFE}_{hmd} + \epsilon_{ihmd} \qquad (1)$$

In equation (1), y_{ihmd} is the outcome observed for patient i visiting ED h on day d of month m. The vector Ind_i includes individual patient characteristics (age, ethnicity, whether lives in a rural area, income deprivation quintile of the LSOA of residence). The vector Att_{imd} includes characteristics of the patient in relation to the ED attendance, namely: mode of arrival, referral source, whether it is a repeated DSH episode, whether the attendance happens in-hours (between 6AM and 6PM) or out-of-hours (between 6PM and 6AM), the volume of ED attendances on the same day. The volume of ED attendances on the same day accounts for differences in the level of pressure faced by ED staff, which may influence their behaviour towards patients. The primary ED diagnosis is used as a proxy for severity of DSH episode (Severity_{ihmd}). This concurs with ambulance arrivals to account for overall patient severity, which may impact how the DSH patient is treated in the ED. The list and frequencies of ED diagnoses is reported in Appendix 1. The vector HDFE represents a high-dimensional fixed effect (HDFE) that includes ED-specific combinations of month of year and day of week, in relation to the day of the ED attendance. The inclusion of a HDFE allows us to account for various supply and demand side observable and unobservable differences across EDs, and days of attendance, that may affect how DSH patients visiting an ED are treated [25]. In absence of more detailed information of staffing at the time of the attendance, this allows to account for shifts in EDs, that are typically arranged well in advance to accommodate known weekly and seasonal fluctuations in ED demand. Similarly, different EDs may have varied organisational structures and staff-skill mix; that is whether the staff are more or less at ease with patients visiting the department for an episode or DSH. Additionally, on the demand side, university students may be more likely to visits EDs for episodes of DSH at a specific time of year (e.g. during end of term exam periods) or location (e.g. hospitals located in major University cities).

Our main coefficient of interest is β associated with the exposure variable Student_i. This reflects the difference in outcomes attributable to being a student according to our proxy definition. In order to interpret β as an unbiased measure of our association of interest, we follow a standard selection on observables argument [26], assuming conditional mean independence. In other words, conditional on the

set of characteristics and the high-dimensional fixed effects we control for, the error term ϵ_{ihmd} is mean independent of our student dummy.

We estimate our models with two related approaches. Firstly, for all outcomes we report estimates of naïve models. These explain outcomes only with our exposure using a Probit model for binary outcomes and a Poisson model for right-skewed outcomes. We then compare our naïve estimates to results from our preferred empirical specification, with and without the more detailed controls for patient severity, represented by primary ED diagnosis. For binary models we assume a linear probability model and estimate equation (1) using a feasible and efficient linear estimator, which accommodates high-dimensional fixed effects [27]. To account for the skewed distribution of waiting times and the number of investigations and procedures, we estimate models for these outcomes using a Poisson regression with a similar feasible efficient estimator designed to accommodate high-dimensional fixed effects [28]. For Poisson models we report results as average marginal effects [29].

Sensitivity analyses

To further explore the relationship between our student proxy and outcomes in ED for DSH attendances we propose two stratified analyses. Firstly, we estimate separately models for attendances during (a) standard in-hours and (b) out-of-hours respectively, as these periods may differ in the level of ED preparedness and resources. Secondly, we estimate two sets of models for (c) patients brought in by ambulance and (d) arrived at the ED by other means. The rationale for this is that patients arriving by ambulance are likely to have a higher priority.

Patient and public involvement

This research did not involve members of the public or patients at any stage, beginning with developing the research questions to identifying the study design or outcomes. We did not consult members of the public in the interpretation of results, nor writing or editing the paper. We also have no specific plans to involve the public in the dissemination of the research findings. Nevertheless, Catherine Campbell developed this research whilst being enrolled as undergraduate student, i.e. as a member of the main target group of this study.

Results

Seasonality of deliberate Self-harm ED attendances

For peer review only

As mentioned in the methods section, there may be seasonality in the attendance of EDs for deliberate self-harm patients. Figure 1 below shows the seasonal variability in the share of patients attending EDs for an episode of deliberate self-harm (averaged across the 127 EDs considered in our analysis).

Figure 1: Share of DSH patients of all ED attendances by month

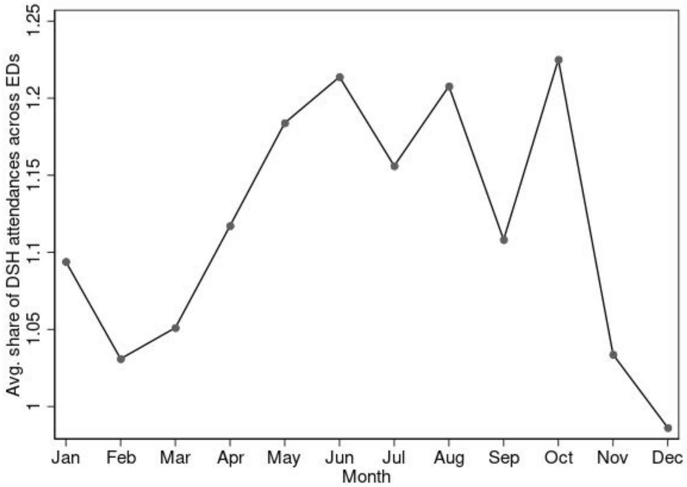

The data suggest a that approximately 1 to 1.25 percent of all monthly ED attendances are related to DSH episodes, with the lowest prevalence of DSH episodes between November and March, and the highest between June and October.

Descriptive statistics

Table 1 below shows characteristics of individuals in the sample, based on our main exposure. There are no substantial differences across covariates between flagged students and non-students. However, we find that a higher percentage of students have repeat attendances and were self-referred, on average. Finally, patients flagged as students are slightly more likely (59 percent compared to 53 percent) to attend the ED during out of hours times (i.e. between 6PM and 6AM and during weekends). This latter characteristic may be relevant for healthcare provision and is hence addressed in the empirical approach.

Table 1: Descriptive statistics for attendances for the full sample and by exposure status

	Full sample		Not flagged as students		Flagged as students	
Variable	Perc.	Count	Perc.	Count	Perc.	Count
Female	59.20	36,944	58.74	34,923	68.46	20,21

For peer review only - http://bmjopen.bmj.com/site/about/guidelines.xhtml

Male	40.80	25,461	41.26	24,530	31.54	931
Aged 10 to 17	15.70	9,802	16.49	9,802		
Aged 18 to 23	21.95	13,696	18.07	10,744	100	2,952
Aged 24 to 29	15.19	9,478	15.94	9,478		
Aged 30 to 34	10.22	6,376	10.72	6,376		
Aged 35 to 39	7.82	4,880	8.21	4,880		
Aged 40 to 44	7.03	4,387	7.38	4,387		
Aged 45 to 49	6.88	4,296	7.23	4,296		
Aged 50 to 54	6.01	3,748	6.3	3,748		
Aged 55 to 59	3.58	2,233	3.76	2,233		
Aged 60 to 64	2.08	1,297	2.18	1,297		
Aged 65 to 69	1.19	740	1.24	740		
Aged 70 plus	2.36	1,472	2.48	1,472		
Ethnicity						
Any White background	94.17	58,766	94.52	56,195	87.09	2,571
Any Black background	1.21	754	1.13	672	2.78	82
Any Indian background	2.16	1,347	2.01	1,193	5.22	154
Any mixed background	2.46	1,538	2.34	1,393	4.91	145
Living in rural area						
Yes	3.08	1,921	3.18	1,891	1.02	30
IMD (income component) deprivation quintile						
Least deprived	10.35	6,459	10.00	5,948	17.31	511
2nd quintile	13.79	8,608	13.97	8,304	10.30	304
3rd quintile	18.82	11,747	18.86	11,215	18.02	532
4th quintile	23.77	14,834	23.92	14,221	20.77	613
Most deprived	33.26	20,757	33.24	19,765	33.60	992
ED arrival mode						
Ambulance	55.85	34,854	56.15	33,383	49.83	1471
Other	43.66	27,249	43.35	25,775	49.93	1474
Not known	0.48	302	0.50	295	0.24	7
Referral source						
Self-referred	47.58	29,690	47.46	28,215	49.97	1,475
GP	1.77	1,106	1.78	1,059	1.59	47
Emergency services	34.22	21,354	34.37	20,432	31.23	922
Health care providers	8.30	5,178	8.28	4,924	8.60	254
Work or education	0.79	490	0.81	481	0.30	9
Other	7.35	4,587	7.30	4,342	8.30	245
Repeated DSH episodes	32.06	20,006	31.91	18,972	35.03	1,034
Out of hours (6AM-6PM) attendance	53.59	33,445	53.28	31,676	59.93	1,769
	Mean (SD)	Range	Mean (SD)	Range	Mean (SD)	Range
Daily ED attendances	410.39 (183.18)	83-1465	408.76 (182.51)	83-1465	443.11 (193.11)	120-1429
Observations	62,405		59,453		2,952	

For peer review only

Table 2: Summary of outcomes by exposure status

	Not flagged as students				Flagged as students			
Discrete outcomes	N	Mean	SD	Range	N	Mean	SD	Range
Time to initial assessment	59,453	17.95	20.64	0-76	2,952	21.24	22.76	0-76
Number of investigations	59,453	3.37	3.45	0-12	2,952	3.13	3.48	0-12
Binary outcomes	N	Perc.	Count		N	Perc.	Count	
Refused or left before treatment	59,453	6.22	3,697		2,952	6.71	198	
Referred to another provider	57,243	45.04	26,776		2,829	35.54	1,049	
Admitted to hospital	59,452	38.20	22,709		2,952	27.98	826	
Discharged with no follow-up	58,857	32.65	19,410		2,923	35.23	1,040	
Unplanned follow-up within 7 days	59,453	5.28	3,138		2,952	5.42	160	

In Table 2 we report summary statistics of outcomes, again for the full sample and by exposure status. The most noticeable difference is that students wait an average of 3.29 minutes longer for their initial assessment, compared to non-students. Moreover, a lower percentage are referred to another provider, indicating there may be differences in care between the two group.

Main Results

Table 3 presents the effects of being a student on several indicators of ED performance, compared to non-students. Results of our naïve model (column 1) suggest an unconditional difference in time to initial assessment of about 3.3 more minutes for DSH patients identified as a student according to our data-based definition (compared to non-students). Although this effect becomes insignificant once we employ our full empirical specification with patient, severity and ED-level covariates. Interestingly, we also find a sizeable reduction in the number of investigations delivered during the visit (7 to 10 percent of the baseline average number of investigations) associated with being flagged as a student. This difference is consistent across the three models in columns 1-3. For patient outcomes and discharge destinations, we find no significant change in the probability of refusing treatment, being discharged with no follow up or having an unplanned follow-up within 7 days. The large and significant decrease in the unconditional probability of being referred to another provider or admitted to hospital compared to other patients (about 10 percent in both cases) is substantially diluted and no longer significant in the fully specified model.

Sensitivity Analysis

For peer review only

In Table 4 we report estimates of the full specification with primary ED diagnosis for our two sensitivity checks, namely the stratified analyses by arrival time (in-hours vs. out-of-hours) and by arrival mode (patients brought in by ambulance vs. arriving at the ED by other means). We find that students attending EDs during in-hours have a 1.8 [0.306, 3.29] percent increased probability of refusing or leaving before treatment, and -0.372 [-0.567,-0.178] less investigations delivered (roughly 10% of baseline average), compared to non-students. DSH patients attending EDs during out-of-hours (weekday nights and weekends), had -0.166 [-0.329,-0.00201] fewer investigations delivered, and had a reduced probability (-2.21 [-3.84,-0.577] of having an unplanned follow-up within 7 days for students compared to non-students.

Columns 3 and 4 reflect the differences in outcomes attributable to whether the patent arrived by ambulance, comparing students and non-students. Whilst the main results in Table 3 account for the mode of arrival, splitting DSH patients by ambulance arrivals may help to better account for an intrinsic unobserved heterogeneity between these patient groups. We find that amongst students who arrive by ambulance, there is a 1.98 [-3.41,0.554] percent lower probability of unplanned follow-up compared to non-students. In addition, we find the number of investigations performed at a patient's initial assessment are again 0.279 [-0.446,-0.112] lower (about 7 percent of the the baseline average number of investigations) among students who do not arrive by ambulance compared to other patients.

Table 4: Stratified analyses by arrival time and arrival mode

		In-hours (1)	Out-of-hours (2)	Arrived by ambulance (3)	Did not arrive by ambulance (4)
Time to initial assessment	*Estimate*	-0.205	0.526	-0.0627	0.427
	95% CI	[-1.474,1.063]	[-0.570,1.622]	[-1.063,0.937]	[-0.964,1.817]
	N	26815	31416	32567	24992
Number of investigations	*Estimate*	-0.372***	-0.166*	-0.0947	-0.279**
	95% CI	[-0.567,-0.178]	[-0.329,-0.00201]	[-0.268,0.0781]	[-0.446,-0.112]
	N	26509	31031	32790	24580
Refused or left before treatment	*Estimate*	0.0180*	-0.000611	0.00706	0.00928
	95% CI	[0.00306,0.0329]	[-0.0151,0.0139]	[-0.0105,0.0246]	[-0.00130,0.0199]
	N	27243	31858	33328	25768
Referred to another provider	*Estimate*	-0.0187	-0.0181	-0.0266	-0.0199
	95% CI	[-0.0552,0.0178]	[-0.0510,0.0147]	[-0.0598,0.00669]	[-0.0583,0.0186]
	N	26102	30588	32029	24662
Admitted to hospital	*Estimate*	-0.0224	-0.0152	-0.0288	-0.00554
	95% CI	[-0.0636,0.0189]	[-0.0475,0.0171]	[-0.0625,0.00478]	[-0.0410,0.0299]
	N	27243	31858	33328	25768
Discharged with no follow-up	*Estimate*	-0.0222	-0.0125	-0.00661	-0.0133
	95% CI	[-0.0496,0.00525]	[-0.0391,0.0141]	[-0.0358,0.0226]	[-0.0429,0.0163]
	N	26968	31495	33047	25420
Unplanned follow-up within 7 days	*Estimate*	-0.00751	-0.0221**	-0.0198**	-0.0105

For peer review only

95% CI	[-0.0272,0.0122]	[-0.0384,-0.00577]	[-0.0341,-0.00554]	[-0.0335,0.0125]
N	27243	31858	33328	25768

Notes: (a) All models and 3 include the following covariates: gender (female or male), age, ethnicity, whether lives in a rural area, income deprivation quintile of the LSOA of residence, mode of arrival, referral source, whether it is a repeated DSH episode, whether the attendance happens in-hours (between 6AM and 6PM) or out-of-hours (between 6PM and 6AM), the volume of ED attendances on the same day at the same ED, primary ED diagnosis. (b) Models were estimated with a Poisson feasible efficient estimator designed to accommodate high-dimensional fixed effects (Correia et al., 2020) for time to initial assessment and number of investigations. For all other outcomes we estimated a linear probability model using a similar feasible efficient linear estimator accommodating high-dimensional fixed effects (Correia, 2016); (c) Estimates represent average marginal effects for the difference between being flagged as students and non-student, 95% confidence intervals in parentheses. Stars indicate statistical significance as follows: * $p < 0.05$, ** $p < 0.01$, *** $p < 0.001$

Discussion

Summary of findings

Our analysis proposes a method to identify patients who are likely students in the HES ED records, and explores differences in healthcare outcomes between students and non-students attending an ED after an episode of deliberate self-harm. We find a statistically significant difference of 0.23 less investigations delivered to students compared to non-students (about 7 percent compared to the baseline number of investigations). All other differences in ED care outcomes are not statistically significant in our main analysis. The results of our stratified analyses by time of attendance (regular times compared to out of hours, i.e. 6PM to 6AM and weekends) and ambulance arrival confirm the main findings providing a more nuanced picture. Patients visiting during regular day hours are slightly more likely to leave before treatment or refuse treatment and receive slightly less investigations. On the other hand, patients visiting during out of hours or arriving by ambulance are less likely to reattend the ED withing 7-days and receive the same amount of investigations as other DSH patients.

Strengths and weaknesses of the study

This paper has several strengths. Firstly, our analysis uses a large dataset capturing ED attendances of DSH patients in English hospitals for an entire year. Secondly, it trials a student proxy indicator to identify and measure differences in emergency care outcomes compared to other patients attending EDs for the same reason – deliberate self-harm. Thirdly, in order to isolate the association between ED care outcomes and being a student, we address potential sources of confounding both on the demand (i.e. patients) and supply (i.e. hospital) sides. To account for compositional differences and unobserved patient severity, our main specification controls for patient characteristics and primary ED diagnosis. On the

For peer review only

supply side, existing literature finds that – based on historical attendance patterns - some variation in attendance volume is predictable and as a result EDs are capable of adjusting capacity accordingly [30]. To circumvent the confounding effect on estimates resulting from ED shift scheduling patterns and differing times of attendance, we control for number of ED attendances on the same day and include an ED-specific high-dimensional fixed effect to clean our estimates from expected patterns of shift planning and natural variations in ED attendance across areas and seasons.

Despite our range of sensitivity checks, some limitations remain. First, our student indicator is only a proxy, and therefore, be interpreted with caution. Nevertheless, our method of identifying students is conservative, suggesting our results are likely interpretable as lower bounds of student attendance volumes and related differences in ED care. Moreover, our study does not include any qualitative component; we do not interview ED staff members in regards to their attitude towards self-harm patients, a practice which would help to interpret some of our findings [12]. Finally, variability in the systems and protocol to assess and treat self-harm presentations may impede a meaningful comparison across areas [31]. We try to circumvent this latter aspect by accounting for hospital-specific fixed effects, and by exploring the heterogeneity in the results across areas more or less exposed to, and equipped for, episodes of deliberate self-harm.

Strengths and weaknesses in relation to other studies

A related study by Chartrand et al. [32] proposes a longitudinal analysis of differences in discharge destination and rate of reattendance between patients who experience DSH and those who attempt suicide. They find those who re-attended for DSH and suicidal attempt were more likely to be discharged to usual care during the initial assessment compared to a patient only attending for DSH. In view of this, a potential weakness of our study – dictated by data limitations – is that we did not include an indicator for suicide attempts. This is an important factor as patients who engage in DSH are at greater risk of subsequently dying by suicide [17]. Our results were consistent with a paper by Sinclair [33] whom employs a crossover methodological design to examine the differences in care before and after the introduction of a dedicated psychiatric nurse service. Importantly, the author finds an increase in the accuracy of assessment and diagnosis post-intervention, wherein nurses are better trained to address DSH

For peer review only

patients during initial assessments. These results may be consistent with a higher effectiveness in assessing patients, and hence explain the presented negative association between patient characteristics (being a student attending for DSH) and the number of investigations.

Substantial effects on the reduced probability of reattending and refusing treatment amongst students emphasises the reality that university students are not a homogenous group; rather there is large variation in health-seeking behaviours within the student population and compared to other patients. Existing literature find that upon arrival at the ED, students experience feelings of shame and fear surrounding a perceived ‘failure to cope’ and lack of confidentiality [34,35]. It is plausible that these feelings are exacerbated when attending in-hours, wherein overcrowding can lead to higher levels of patients incorrectly leaving the ED without treatment when care was required [36].

The decreased number of investigations amongst students, compared to non-students, is consistent with physician’s actions being guided by a both a priority to treat physical symptoms (over psychological symptoms) and limited knowledge in terms of the immediate management of self-harm patients. In particular, qualitative literature finds self-harm patients experience negative attitudes from staff and differences in treatment compared to other patients [37]. These patterns may originate from an increased level of uncertainty in dealing with this patient group, wherein a deficit in self-harm training has be emphasised [35]. Previous literature has also raised concerns over a lack of local policies that prioritise mental health over physical health [38].

In addition, the large and significant reduction in the number of investigations for patients who do not arrive via ambulance may reflect faults in the ED triage process. The first step upon arrival requires patients to provide a brief summary of their health condition(s) at reception. Following this, patients wait for an initial assessment, wherein a medical or nursing professional determines priority for treatment by taking a brief medical history, a pain assessment and any collection of early warning scores [39]. Therefore, if staff are not trained in the management of mental health patients, EDs overlook a crucial opportunity to diagnose mental illness and commence a treatment pathway that may prevent subsequent self-harming behaviour [40].

For peer review only

Policy Implications

Poor mental health is a major clinical priority given that it is at least partially preventable and a contributing risk factor for suicide attempts, especially among teens and young adults [41]. Our results have implications for both university health care and ED systems. English universities need to further invest in resources to address the heightened demands for counselling services [42]. Oversubscription and a shortage of trained staff have impacted services through reduced length and number of sessions, longer waitlists, and more referrals to off-campus providers [2]. In addition, university health centres need to prioritise information campaigns to decrease the stigma surrounding self-harm that prevents many students from seeking help, and consequently, lead to EDs being a first point of contact. The higher probability of refusing or leaving without treatment when attending EDs in-hours suggests the need for greater access to dedicated mental health staff or treatment, better access to community-based resources, increased availability of inpatient psychiatric beds and a separate safe space in the ED [43]. In addition, roughly one half of DSH patients in ED do not receive a psychosocial assessment [44], which further indicates the need to regulate and standardise mental health services. This suggest that better coordination between university mental health services and EDs may be needed in the long-term. In the short-term, in view of existing time and resource constraints, educating existing staff in the evaluation and management of DSH patients may be the only feasible approach to address such issues.

Open questions and future research

This paper could be advanced in several ways. Further research is needed to investigate the association between students and ED outcomes amongst those who have current contact with mental health services at the time of self-harm. Future studies should aim to understand differences in student's health-seeking behaviour that could help design policies to mitigate staff bias towards this sub-group. Future research should also focus on the scarcity of integrated inpatient and outpatient mental health services at the county level. This is crucial in providing unified health care for a high-risk and vulnerable population, whilst also reducing capacity constraints on emergency departments.

Declarations

For peer review only

Authors' contributions: CC and IF developed the original idea for the study, designed the analysis and carried out the analysis. CC, IF and JD contributed substantially to the interpretation of results and the writing of the manuscript. IF is responsible for the overall content as the guarantor.

Funding: This research was supported by the National Institute for Health Research (NIHR) Applied Research Collaboration Greater Manchester (NIHR200174).

Competing interests: None to declare

Data availability statement: The Hospital Episode Statistics data that support the findings of this study are available from NHS Digital. Restrictions apply to the availability of these data. They were used under licence for this study, and so are not publicly available.

Patient consent for publication: Not applicable.

Ethics approval: Not applicable.

Acknowledgments: We are grateful for comments and suggestions received from colleagues during Health Organisation, Policy and Economics (HOPE) seminars at the University of Manchester. Additional thanks to the Winter 2022 Health Economics Study Group (HESG) conference participants in Leeds for feedback during the poster session.

For peer review only

References

1 Storrie K, Ahern K, Tuckett A. A systematic review: Students with mental health problems—A growing problem. *Int J Nurs Pract* 2010;**16**:1–6. doi:10.1111/j.1440-172X.2009.01813.x

2 Gallagher RP. Thirty Years of the National Survey of Counseling Center Directors: A Personal Account. *J Coll Stud Psychother* 2012;**26**:172–84. doi:10.1080/87568225.2012.685852

3 Kessler RC, Amminger GP, Aguilar-Gaxiola S, *et al.* Age of onset of mental disorders: a review of recent literature. *Curr Opin Psychiatry* 2007;**20**:359–64. doi:10.1097/YCO.0b013e32816ebc8c

4 Dodd AL. Student mental health research: moving forwards with clear definitions. *J Ment Health* 2021;**30**:273–5. doi:10.1080/09638237.2020.1844873

5 Gunnell D, Caul S, Appleby L, *et al.* The incidence of suicide in University students in England and Wales 2000/2001–2016/2017: Record linkage study. *J Affect Disord* 2020;**261**:113–20. doi:10.1016/j.jad.2019.09.079

6 Macaskill A. The mental health of university students in the United Kingdom. *Br J Guid Couns* 2013;**41**:426–41. doi:10.1080/03069885.2012.743110

7 McManus S, Gunnell D. Trends in mental health, non-suicidal self-harm and suicide attempts in 16–24-year old students and non-students in England, 2000–2014. *Soc Psychiatry Psychiatr Epidemiol* 2020;**55**:125–8. doi:10.1007/s00127-019-01797-5

8 Querstret D. Collaborating with Students to Support Student Mental Health and Well-being. In: Lygo-Baker S, Kinchin IM, Winstone NE, eds. *Engaging Student Voices in Higher Education : Diverse Perspectives and Expectations in Partnership*. Cham: : Springer International Publishing 2019. 191–207. doi:10.1007/978-3-030-20824-0_12

9 Wilson C, Spencer G. The mental health support needs of university students with long-term physical health conditions. *Health Educ* 2022;**122**:584–98. doi:10.1108/HE-01-2022-0004

10 Yorgason JB, Roper SO, Sandberg JG, *et al.* Stress spillover of health symptoms from healthy spouses to patient spouses in older married couples managing both diabetes and osteoarthritis. *Fam Syst Health* 2012;**30**:330–43.

11 Mackay N, Barrowclough C. Accident and emergency staff's perceptions of deliberate self-harm: Attributions, emotions and willingness to help. *Br J Clin Psychol* 2005;**44**:255–67. doi:10.1348/014466505X29620

12 Cleaver K. Attitudes of emergency care staff towards young people who self-harm: A scoping review. *Int Emerg Nurs* 2014;**22**:52–61. doi:10.1016/j.ienj.2013.04.001

For peer review only

13 Timson D, Priest H, Clark-Carter D. Adolescents who self-harm: Professional staff knowledge, attitudes and training needs. *J Adolesc* 2012;**35**:1307–14. doi:10.1016/j.adolescence.2012.05.001

14 Holt M, Powell S. Healthy Universities: a guiding framework for universities to examine the distinctive health needs of its own student population. *Perspect Public Health* 2017;**137**:53–8. doi:10.1177/1757913916659095

15 Tran D-MT, Silvestri-Elmore A. Healthcare-seeking behaviours in college students and young adults: a review. *J Res Nurs* 2021;**26**:320–38. doi:10.1177/1744987120951594

16 Beckman K, Mittendorfer-Rutz E, Waern M, *et al.* Method of self-harm in adolescents and young adults and risk of subsequent suicide. *J Child Psychol Psychiatry* 2018;**59**:948–56. doi:10.1111/jcpp.12883

17 Bergen H, Hawton K, Waters K, *et al.* How do methods of non-fatal self-harm relate to eventual suicide? *J Affect Disord* 2012;**136**:526–33. doi:10.1016/j.jad.2011.10.036

18 Chan MKY, Bhatti H, Meader N, *et al.* Predicting suicide following self-harm: systematic review of risk factors and risk scales. *Br J Psychiatry* 2016;**209**:277–83. doi:10.1192/bjp.bp.115.170050

19 Hawton K, Bergen H, Kapur N, *et al.* Repetition of self-harm and suicide following self-harm in children and adolescents: findings from the Multicentre Study of Self-harm in England. *J Child Psychol Psychiatry* 2012;**53**:1212–9. doi:10.1111/j.1469-7610.2012.02559.x

20 Department for Communities and Local Government. English indices of deprivation 2015. 2015. https://www.gov.uk/government/statistics/english-indices-of-deprivation-2015

21 NHS Digital. Patients Registered at a GP Practice. NHS Digit. 2022.https://digital.nhs.uk/data-and-information/publications/statistical/patients-registered-at-a-gp-practice (accessed 19 Aug 2022).

22 Paul E, Ortin A. Correlates of Suicidal Ideation and Self-harm in Early Childhood in a Cohort at Risk for Child Abuse and Neglect. *Arch Suicide Res* 2019;**23**:134–50. doi:10.1080/13811118.2017.1413468

23 The King's Fund. What's going on with A&E waiting times? 2020.https://www.kingsfund.org.uk/projects/urgent-emergency-care/urgent-and-emergency-care-mythbusters (accessed 11 May 2022).

24 Anselmi L, Everton A, Shaw R, *et al.* Estimating local need for mental healthcare to inform fair resource allocation in the NHS in England: cross-sectional analysis of national administrative data linked at person level. *Br J Psychiatry* 2020;**216**:338–44. doi:10.1192/bjp.2019.185

For peer review only

25 Turner AJ, Anselmi L, Lau Y-S, *et al.* The effects of unexpected changes in demand on the performance of emergency departments. *Health Econ* 2020;**29**:1744–63. doi:https://doi.org/10.1002/hec.4167

26 Altonji JG, Elder TE, Taber CR. Selection on Observed and Unobserved Variables: Assessing the Effectiveness of Catholic Schools. *J Polit Econ* 2005;**113**:151–84. doi:10.1086/426036

27 Correia S. A Feasible Estimator for Linear Models with Multi-Way Fixed Effects. 2016. http://scorreia.com/research/hdfe.pd

28 Correia S, Guimarães P, Zylkin T. Fast Poisson estimation with high-dimensional fixed effects. *Stata J* 2020;**20**:95–115. doi:10.1177/1536867X20909691

29 Norton EC, Dowd BE, Maciejewski ML. Marginal Effects—Quantifying the Effect of Changes in Risk Factors in Logistic Regression Models. *JAMA* 2019;**321**:1304–5. doi:10.1001/jama.2019.1954

30 Anselmi L, Meacock R, Kristensen SR, *et al.* Arrival by ambulance explains variation in mortality by time of admission: retrospective study of admissions to hospital following emergency department attendance in England. *BMJ Qual Saf* 2017;**26**:613–21. doi:10.1136/bmjqs-2016-005680

31 Joyce M, Chakraborty S, O'Sullivan G, *et al.* National Self-Harm Registry Ireland Annual Report 2020. Cork: : National Suicide Research Foundation 2022.

32 Chartrand H, Tefft B, Sareen J, *et al.* A Longitudinal Study of Correlates, Discharge Disposition, and Rate of Re-presentation to Emergency Services of Adults who Engage in Non-suicidal Self-injury. *Arch Suicide Res* 2022;**26**:1141–58. doi:10.1080/13811118.2020.1856259

33 Sinclair L. How effective are mental health nurses in A&E departments? *Emerg Med J* 2006;**23**:687–92. doi:10.1136/emj.2005.033175

34 Owens C, Hansford L, Sharkey S, *et al.* Needs and fears of young people presenting at accident and emergency department following an act of self-harm: secondary analysis of qualitative data. *Br J Psychiatry* 2016;**208**:286–91. doi:10.1192/bjp.bp.113.141242

35 Mulhearne P, Cotter P, O'Shea M, *et al.* Experiences of registered general nurses who care for patients presenting with self-harm to the emergency department in Ireland. *Int Emerg Nurs* 2021;**58**:101047. doi:10.1016/j.ienj.2021.101047

36 Miró Ò, Antonio MT, Jiménez S, *et al.* Decreased health care quality associated with emergency department overcrowding: *Eur J Emerg Med* 1999;**6**:105–7. doi:10.1097/00063110-199906000-00003

37 Gibson R, Carson J, Houghton T. Stigma towards non-suicidal self-harm: evaluating a brief educational intervention. *Br J Nurs* 2019;**28**:307–12. doi:10.12968/bjon.2019.28.5.307

For peer review only

38 Wolf LA, Perhats C, Delao AM. US emergency nurses' perceptions of challenges and facilitators in the management of behavioural health patients in the emergency department: A mixed-methods study. *Australas Emerg Nurs J* 2015;**18**:138–48. doi:10.1016/j.aenj.2015.03.004

39 Department of Health. A&E Clinical Quality Indicators. 2010. https://webarchive.nationalarchives.gov.uk/ukgwa/20130105030902/http://www.dh.gov.uk/en/Publicationsandstatistics/Publications/PublicationsPolicyAndGuidance/DH_122868

40 Butler C, Joiner R, Bradley R, *et al.* Self-harm prevalence and ideation in a community sample of cis, trans and other youth. *Int J Transgenderism* 2019;**20**:447–58. doi:10.1080/15532739.2019.1614130

41 Furber G, Segal L, Leach M, *et al.* Preventing mental illness: closing the evidence-practice gap through workforce and services planning. *BMC Health Serv Res* 2015;**15**:283. doi:10.1186/s12913-015-0954-5

42 Prince JP. University student counseling and mental health in the United States: Trends and challenges. *Ment Health Prev* 2015;**3**:5–10. doi:10.1016/j.mhp.2015.03.001

43 Cullen SW, Diana A, Olfson M, *et al.* If You Could Change 1 Thing to Improve the Quality of Emergency Care for Deliberate Self-harm Patients, What Would It Be? A National Survey of Nursing Leadership. *J Emerg Nurs* 2019;**45**:661–9. doi:10.1016/j.jen.2019.06.007

44 Bennewith O, Peters TJ, Hawton K, *et al.* Factors associated with the non-assessment of self-harm patients attending an Accident and Emergency Department: Results of a national study. *J Affect Disord* 2005;**89**:91–7. doi:10.1016/j.jad.2005.08.011

For peer review only

Appendix 1: Distribution of primary ED codes (severity proxy) for analytical sample of attendances

	Freq.	Percent	Cum.
1 Laceration	4,659	7.47	7.47
2 Contusion/abrasion	1,595	2.56	10.02
3 Soft tissue inflammation	484	0.78	10.8
4 Head injury	532	0.85	11.65
5 Dislocation/fracture/joi	1,189	1.91	13.56
6 Sprain/ligament injury	508	0.81	14.37
7 Muscle/tendon injury	165	0.26	14.63
8 Nerve injury	10	0.02	14.65
9 Vascular injury	7	0.01	14.66
10 Burns and scalds	171	0.27	14.93
11 Electric shock	7	0.01	14.95
12 Foreign body	760	1.22	16.16
13 Bites/stings	35	0.06	16.22
14 Poisoning (inc overdose)	21,744	34.84	51.06
15 Near drowning	12	0.02	51.08
16 Visceral injury	23	0.04	51.12
17 Infectious disease	47	0.08	51.19
18 Local infection	154	0.25	51.44
19 Septicaemia	28	0.04	51.49
20 Cardiac conditions	212	0.34	51.83
21 Cerebro-vascular conditions	48	0.08	51.9
22 Other vascular conditions	38	0.06	51.96
23 Haematological conditions	27	0.04	52.01
24 Central nervous system conditions (exc stroke)	209	0.33	52.34
25 Respiratory conditions	127	0.2	52.55
26 Gastrointestinal conditions	310	0.5	53.04
27 Urological conditions (inc cystitis)	101	0.16	53.2
28 Obstetric conditions	7	0.01	53.22
29 Gynaecological conditions	149	0.24	53.45
30 Diabetes and other endocrinological conditions	71	0.11	53.57
31 Dermatological conditions	22	0.04	53.6
32 Allergy (inc anaphylaxis)	21	0.03	53.64
33 Facio-maxillary conditions	15	0.02	53.66
34 ENT conditions	54	0.09	53.75
35 Psychiatric conditions	9,156	14.67	68.42
36 Ophthalmological conditions	135	0.22	68.64
37 Social problems (inc chronic alcoholism and homelessness)	661	1.06	69.69
38 Diagnosis not classifiable	6,333	10.15	79.84
39 Nothing abnormal detected	881	1.41	81.25
40 Missclassified	2,803	4.49	85.75
41 No/missing diagnosis	8,895	14.25	100
Total	62,405	100	

For peer review only

The RECORD statement – checklist of items, extended from the STROBE statement, that should be reported in observational studies using routinely collected health data.

	Item No.	STROBE items	Location in manuscript where items are reported	RECORD items	Location in manuscript where items are reported
**Title and abstract**					
	1	(a) Indicate the study's design with a commonly used term in the title or the abstract (b) Provide in the abstract an informative and balanced summary of what was done and what was found	Title and abstract – page 1.	RECORD 1.1: The type of data used should be specified in the title or abstract. When possible, the name of the databases used should be included.<br><br>RECORD 1.2: If applicable, the geographic region and timeframe within which the study took place should be reported in the title or abstract.<br><br>RECORD 1.3: If linkage between databases was conducted for the study, this should be clearly stated in the title or abstract.	Title and abstract – page 1.
**Introduction**					
Background rationale	2	Explain the scientific background and rationale for the investigation being reported	Introduction – page 1.		
Objectives	3	State specific objectives, including any prespecified hypotheses	Introduction, final paragraph – pages 1 & 2.		
**Methods**					
Study Design	4	Present key elements of study design early in the paper	Methods – pages 2-4.		
Setting	5	Describe the setting, locations, and relevant dates, including periods of recruitment, exposure, follow-up, and data collection	Methods, subsections: 'data and sample' (page 2) and 'exposure' (page 3).		

For peer review only

Participants	6	*(a) Cohort study* - Give the eligibility criteria, and the sources and methods of selection of participants. Describe methods of follow-up<br>*Case-control study* - Give the eligibility criteria, and the sources and methods of case ascertainment and control selection. Give the rationale for the choice of cases and controls<br>*Cross-sectional study* - Give the eligibility criteria, and the sources and methods of selection of participants<br><br>*(b) Cohort study* - For matched studies, give matching criteria and number of exposed and unexposed<br>*Case-control study* - For matched studies, give matching criteria and the number of controls per case	Methods, subsection 'data and sample' – page 2.	RECORD 6.1: The methods of study population selection (such as codes or algorithms used to identify subjects) should be listed in detail. If this is not possible, an explanation should be provided.<br><br>RECORD 6.2: Any validation studies of the codes or algorithms used to select the population should be referenced. If validation was conducted for this study and not published elsewhere, detailed methods and results should be provided.<br><br>RECORD 6.3: If the study involved linkage of databases, consider use of a flow diagram or other graphical display to demonstrate the data linkage process, including the number of individuals with linked data at each stage.	Methods, subsection 'data and sample' – page 2.
Variables	7	Clearly define all outcomes, exposures, predictors, potential confounders, and effect modifiers. Give diagnostic criteria, if applicable.	Methods, subsections 'exposure', 'outcomes' and 'empirical approach' – pages 3 & 4.	RECORD 7.1: A complete list of codes and algorithms used to classify exposures, outcomes, confounders, and effect modifiers should be provided. If these cannot be reported, an explanation should be provided.	Methods, subsection 'empirical approach' – pages 3 & 4.
Data sources/ measurement	8	For each variable of interest, give sources of data and details of methods of assessment (measurement).<br>Describe comparability of assessment methods if there is more than one group	Methods – pages 2-4.		

For peer review only

Bias	9	Describe any efforts to address potential sources of bias	Methods, subsection 'empirical approach' – page 4.		
Study size	10	Explain how the study size was arrived at	Methods, subsections 'data and sample' and 'exposure' – pages 2 & 3.		
Quantitative variables	11	Explain how quantitative variables were handled in the analyses. If applicable, describe which groupings were chosen, and why	Methods, subsection 'empirical approach' – pages 3 & 4.		
Statistical methods	12	(a) Describe all statistical methods, including those used to control for confounding<br>(b) Describe any methods used to examine subgroups and interactions<br>(c) Explain how missing data were addressed<br>(d) *Cohort study* - If applicable, explain how loss to follow-up was addressed<br>*Case-control study* - If applicable, explain how matching of cases and controls was addressed<br>*Cross-sectional study* - If applicable, describe analytical methods taking account of sampling strategy<br>(e) Describe any sensitivity analyses	Methods, subsections 'empirical approach' and 'sensitivity analyses' – pages 3 & 4.		
Data access and cleaning methods		..		RECORD 12.1: Authors should describe the extent to which the investigators had access to the database	Methods, subsection 'data and sample' – page 2.

For peer review only - http://bmjopen.bmj.com/site/about/guidelines.xhtml

				population used to create the study population.<br><br>RECORD 12.2: Authors should provide information on the data cleaning methods used in the study.	
Linkage		..		RECORD 12.3: State whether the study included person-level, institutional-level, or other data linkage across two or more databases. The methods of linkage and methods of linkage quality evaluation should be provided.	Methods, subsection 'data and sample' – page 2.
**Results**					
Participants	13	(a) Report the numbers of individuals at each stage of the study (*e.g.*, numbers potentially eligible, examined for eligibility, confirmed eligible, included in the study, completing follow-up, and analysed)<br>(b) Give reasons for non-participation at each stage.<br>(c) Consider use of a flow diagram	Methods, subsections 'data and sample' and 'exposure' – pages 2 & 3.	RECORD 13.1: Describe in detail the selection of the persons included in the study (*i.e.,* study population selection) including filtering based on data quality, data availability and linkage. The selection of included persons can be described in the text and/or by means of the study flow diagram.	Methods, subsections 'data and sample' and 'exposure' – pages 2 & 3.
Descriptive data	14	(a) Give characteristics of study participants (*e.g.*, demographic, clinical, social) and information on exposures and potential confounders<br>(b) Indicate the number of participants with missing data for each variable of interest<br>(c) *Cohort study* - summarise follow-up time (*e.g.*, average and total amount)	Results, subsection 'descriptive statistics' – pages 5 & 6.		

For peer review only

Outcome data	15	*Cohort study* - Report numbers of outcome events or summary measures over time<br>*Case-control study* - Report numbers in each exposure category, or summary measures of exposure<br>*Cross-sectional study* - Report numbers of outcome events or summary measures	Results, subsection 'descriptive statistics' – pages 7.		
Main results	16	(a) Give unadjusted estimates and, if applicable, confounder-adjusted estimates and their precision (e.g., 95% confidence interval). Make clear which confounders were adjusted for and why they were included<br>(b) Report category boundaries when continuous variables were categorized<br>(c) If relevant, consider translating estimates of relative risk into absolute risk for a meaningful time period	Results, subsection 'main results' – pages 7.		
Other analyses	17	Report other analyses done—e.g., analyses of subgroups and interactions, and sensitivity analyses	Results, subsection 'sensitivity analyses' – pages 7 & 8.		
**Discussion**					
Key results	18	Summarise key results with reference to study objectives	Discussion, subsection 'summary of findings' – page 8 & 9.		
Limitations	19	Discuss limitations of the study, taking into account sources of potential bias or imprecision.	Discussion – pages 9 & 10.	RECORD 19.1: Discuss the implications of using data that were not created or collected to answer the specific research question(s). Include	Discussion – pages 9 & 10.

For peer review only

		Discuss both direction and magnitude of any potential bias		discussion of misclassification bias, unmeasured confounding, missing data, and changing eligibility over time, as they pertain to the study being reported.	
Interpretation	20	Give a cautious overall interpretation of results considering objectives, limitations, multiplicity of analyses, results from similar studies, and other relevant evidence	Discussion, pages 8-10.		
Generalisability	21	Discuss the generalisability (external validity) of the study results			
**Other Information**					
Funding	22	Give the source of funding and the role of the funders for the present study and, if applicable, for the original study on which the present article is based	Declarations, page 12.		
Accessibility of protocol, raw data, and programming code		..		RECORD 22.1: Authors should provide information on how to access any supplemental information such as the study protocol, raw data, or programming code.	

*Reference: Benchimol EI, Smeeth L, Guttmann A, Harron K, Moher D, Petersen I, Sørensen HT, von Elm E, Langan SM, the RECORD Working Committee. The REporting of studies Conducted using Observational Routinely-collected health Data (RECORD) Statement. *PLoS Medicine* 2015; in press.

*Checklist is protected under Creative Commons Attribution (CC BY) license.

For peer review only

BMJ Open

Outcomes for university students following emergency care presentation for deliberate self-harm: retrospective observational study of Emergency Departments in England for 2017/2018

Journal:	*BMJ Open*
Manuscript ID	bmjopen-2023-078672.R1
Article Type:	Original research
Date Submitted by the Author:	09-Jan-2024
Complete List of Authors:	Campbell, Catherine; The University of Manchester, Centre for Primary Care and Health Services Research<br>Dodd, Joe; The University of Manchester, Centre for Primary Care and Health Services Research<br>Francetic, Igor; The University of Manchester, Centre for Primary Care and Health Services Research
**Primary Subject Heading**:	Emergency medicine
Secondary Subject Heading:	Mental health
Keywords:	Suicide & self-harm < PSYCHIATRY, ACCIDENT & EMERGENCY MEDICINE, Patients, MENTAL HEALTH

SCHOLARONE™
Manuscripts

I, the Submitting Author has the right to grant and does grant on behalf of all authors of the Work (as defined in the below author licence), an exclusive licence and/or a non-exclusive licence for contributions from authors who are: i) UK Crown employees; ii) where BMJ has agreed a CC-BY licence shall apply, and/or iii) in accordance with the terms applicable for US Federal Government officers or employees acting as part of their official duties; on a worldwide, perpetual, irrevocable, royalty-free basis to BMJ Publishing Group Ltd ("BMJ") its licensees and where the relevant Journal is co-owned by BMJ to the co-owners of the Journal, to publish the Work in this journal and any other BMJ products and to exploit all rights, as set out in our licence.

The Submitting Author accepts and understands that any supply made under these terms is made by BMJ to the Submitting Author unless you are acting as an employee on behalf of your employer or a postgraduate student of an affiliated institution which is paying any applicable article publishing charge ("APC") for Open Access articles. Where the Submitting Author wishes to make the Work available on an Open Access basis (and intends to pay the relevant APC), the terms of reuse of such Open Access shall be governed by a Creative Commons licence – details of these licences and which Creative Commons licence will apply to this Work are set out in our licence referred to above.

Other than as permitted in any relevant BMJ Author's Self Archiving Policies, I confirm this Work has not been accepted for publication elsewhere, is not being considered for publication elsewhere and does not duplicate material already published. I confirm all authors consent to publication of this Work and authorise the granting of this licence.

For peer review only

Title: Outcomes for university students following emergency care presentation for deliberate self-harm: retrospective observational study of Emergency Departments in England for 2017/2018

Authors: Catherine Campbell, Research Assistant [a], Joe Dodd [a], Research Associate, Igor Francetic, Research Fellow [a]

Affiliation: [a] Health Organisation, Policy and Economics (HOPE) group, School of Health Sciences, University of Manchester, UK.

 Corresponding author: Igor Francetic, Health Organisation, Policy and Economics (HOPE) group, School of Health Sciences, University of Manchester, Williamson Building, Oxford Road, M13 9PL, Manchester, UK. Email: igor.francetic@manchester.ac.uk.

Abstract

- **Objectives:** Identify university-aged students and contrast their healthcare provision and outcomes with other patients in the same age group attending emergency departments for deliberate self-harm.
- **Design:** Retrospective cross-sectional observational study.
- **Setting:** Patients visiting 129 public hospital Emergency Departments across England between April 2017 and March 2018.
- **Participants:** 14,074 patients aged 18-23 visiting Emergency Departments for conditions linked to deliberate self-harm, 1,016 of which were identified as university-aged students.
- **Primary and secondary outcome measures:** We study various outcomes across the entire patient pathway in the Emergency Department: Waiting time to initial assessment upon arrival at the emergency department, count of investigations delivered, discharge destination (patients refusing treatment or leave before being seen, referred to another provider or admitted to inpatient care, discharged with no follow-up) and unplanned follow-up visit within 7 days.
- **Results:** We find a statistically significant difference of 0.262 [-0.491,-0.0327] less investigations delivered to students compared to non-students (about 8 percent compared to the baseline number of investigations for non-students). Stratified analyses reveal that this difference is concentrated amongst students visiting the Emergency Department outside of regular working hours (-0.485 [-0.850,-0.120]) and students visiting for repeated deliberate self-harm episodes (-0.881 [-1.510,-0.252]). Unplanned reattendance within 7 days is lower among students visiting Emergency Departments during out of hours (-0.0306 [-0.0576,-0.00363]), whilst students arriving by ambulance are less likely to be referred to another provider (-0.0708 [-0.140,-0.00182]) compared to non-students.
- **Conclusions**: We find evidence of less intense investigations being delivered to patients aged 18-23 identified as students compared to non-students visiting Emergency Departments after an episode of deliberate self-harm. Given the high risk of suicide attempts after episodes of deliberate self-harm among students, our findings may highlight the need for more focused interventions on this group of patients.

Version: January 2024

For peer review only

Word count: 4,445

Strengths and limitations of this study

- We propose an approximate approach to identify students in administrative records for Emergency Departments in England, focusing on the population of attendances between April 2017 and March 2018.
- Supply-side confounders are accounted by a high-dimensional fixed effects, accounting for Emergency Department specific combinations of day of the week and month of the year.
- We study outcomes across the entire pathway for patients attending Emergency Departments after episodes of deliberate self-harm, comparing students and non-students.

For peer review only

Title: Outcomes for university students following emergency care presentation for deliberate self-harm: retrospective observational study of Emergency Departments in England for 2017/2018

Introduction

Three-quarters of mental health disorders peak by the age of 25 years, an age-group associated with university attendance [1] and for which there is a large unmet need for mental health care [2]. As a result, there have been calls for increased research on student mental health [3]. Academic pressures and living far from social networks developed during early adolescence may pose additional stress on an already vulnerable group [4]. For instance, in the U.S, among 274 surveyed universities, 88 percent of counselling centre's directors reported an increase in severe psychological problems over the previous five years [5]. However, in England, trends in incidence of severe mental health problems and suicide do not seem to be higher among students compared to the general population of the same age [6–8]. Nevertheless, from a service provision perspective, the student population appears particularly vulnerable, especially in regards to limited availability and accessibility of mental health services for students [9–11].

Our study relates to literature surrounding the quality of care within emergency departments (EDs) for deliberate self-harm patients. Firstly, ED staff attitude can exert a crucial effect on the future health-seeking behaviours of self-harm patients [12]. This suggests that if a patient experiences a negative reaction to seeking help for their ailment, they are less likely to seek similar help again. Moreover, there is evidence patients who engage in self-harm or attempt suicide are found to be more negatively evaluated and viewed as "wasting resources" compared to patients attending for other reasons [13]. Timson et al. [14] further find a correlation between staff knowledge of mental health treatment and negativity towards self-harm patients. Finally, there is ample evidence relating hospital attendance for non-fatal self-harm to subsequent risk of completed and attempted suicide, indicating that treatment for self-harm attendances is particularly sensitive [15–18]. These studies highlight the potential impact of staff attitudes and perception of mental health on care outcomes for students attending ED for deliberate self-harm.

This paper builds on a growing body of literature regarding student mental health at university. Bolotnyy et al. [19] find that PhD economic students at top-ranked American universities have experienced depressive symptoms at a rate of over two times the population average, with only a small percentage receiving help. Levels of depressive symptoms are even higher among graduate students attending European universities [20]. These studies demonstrate the prevalence and severity of mental health in universities, common across countries. As mentioned, demand-side barriers such as misperceptions about care-seeking may contribute to the treatment gap among university students [21,22]. In light of this, Acampora et al. [23] find that amongst individuals who receive information about the benefits of seeking help, females present a higher likelihood of seeking help, resulting in improved mental health scores at follow-up. Holt and Powell [24] further find that 80 percent of university student attendees are not registered with a GP. This may reflect in an apparent misuse of emergency care services, which has been attributed to students failing to seek healthcare on campus due to fear of vulnerability and breached confidentiality [25].

Building on these stylised facts, we aim to explicitly study the healthcare provision and outcomes of university-aged students attending emergency departments for deliberate self-harm (DSH), contrasting them with other DSH patients. In doing so, given that administrative hospital records do not include information about student status, we propose a proxy indicator to identify students in Hospital Episode Statistics (HES) records.

For peer review only - http://bmjopen.bmj.com/site/about/guidelines.xhtml

Methods

Data and sample

Our study builds on the attendance records of patients visiting Emergency Departments across England between April 2017 and March 2018. These records were obtained from the Hospital Episode Statistics (HES) [26]. HES contains routinely collected data covering procedures and treatments for all patients visiting secondary care facilities across England, tracking the patients' pathway from attendance to discharge. Attendance records contain an extensive range of information about patients, including the hospital site, admission and discharge dates and times, GP practice to which the patient is registered, and a comprehensive set of demographic information. HES also captures the patient's small geographical area of residence. This is at the Lower layer Super Output Area (LSOAs) level, which includes approximately 1,500 individuals. These are used to link information on area-level of deprivation, namely the income component of the Index of Multiple Deprivation [27].

Our target population is represented by patients aged 18 to 23 visiting EDs in England between April 2017 and March 2018, whose attendance was related to an episode of DSH (n=22,506). We focus on this period because of limitations in access to more recent data. Incidentally, this protects our analysis from any disruptions to services caused by the COVID-19 pandemic.

To obtain our analytical sample, we exclude attendances without a recorded LSOA (n=748). We also exclude records with missing values on key outcome or control variables (n=6,349), and with implausible waiting time values (zero total attendance time or with waiting times recorded at the maximum value allowed by the system, n=507). To avoid ambiguity, we also exclude the few patients appearing more than once on the same day in the dataset (n=115). Furthermore, we exclude patients attending Type 2 or Type 3 EDs (n=713), hence focusing only on visits to Type 1 EDs; that is 24-hour consultant-led services with full facilities for resuscitating patients. We do this as Type 2 and Type 3 EDs tend to have a different and less severe mix of patients [28]. Our final analytical sample is composed by 14,074 attendances categorised as related to episodes of deliberate self-harm, across 127 EDs in England, between April 2017 and March 2018. In Table 1 below we show descriptive statistics of our main covariates for the entire sample and for the two subgroups defined by our exposure.

For peer review only

From the Office for National Statistics, we obtain data on the demographic structure of LSOAs. We use this to identify areas with a disproportionately higher share of individuals aged 18-25, an age-range consistent with being a university student. Appendix 1 shows that the geographic distribution of these areas is aligned with the location of universities in England. From regular NHS Digital public releases [29] we obtained the number of patients registered with GP practices divided in five-year age groups. For each GP practice we computed the share of young patients aged between 14 and 44.

Exposure: Proxy indicator for university students visiting EDs in England

Our main exposure is represented by a proxy indicator which aims to identify university students visiting an ED in England based on HES Accident and Emergency (A&E) records. This proxy attempts to circumvent the absence of a specific variable indicating whether a patient is a student. Firstly, we define 18 to 23 as our target age range consistent with most university students. Secondly, we identify LSOAs where more than 10 percent of residents are aged between 18 and 25. This second criterion tries to identify areas in England where student residences are located (see Appendix 1). Thirdly, we identify GP practices whose patients are predominantly within an age range consistent with university students [30]. Specifically, we flagged 1,016 DSH attendance from patients aged 18 to 23 living in LSOAs likely to host students and registered with GP practices with over 50 percent of patients aged 14 to 44 (hence likely to treat students). This is equivalent to 7.22 percent of our sample.

Outcomes

Our analysis aims to study the healthcare provision and outcomes of university-aged students attending emergency departments for deliberate self-harm, contrasting them with other DSH patients in the same age range. The outcomes studied cover the entire patient pathway in ED from arrival to discharge. However, we do not focus on the choice of specific treatments delivered and their intensity as these are likely to depend on patient severity, which we are unable to measure. We firstly look at the time to initial assessment upon arrival at the ED. Secondly, we look at the count of investigations delivered. Thirdly, we look at various discharge destinations: patients refusing treatment or leave before being seen, referred to another provider or admitted to inpatient care, discharged with no follow-up. Finally, we measure unplanned follow-up visits within 7 days.

For peer review only

Empirical approach

A simple comparison in means across groups of patients by student status is unlikely to give an accurate account of differences in outcomes due to various sources of confounding. For example, patients flagged as students may have more or less severe conditions than other DSH patients. They may also systematically visit EDs that are intrinsically different or at times where EDs are more or less busy or staffed. To study whether being a student is associated with differences in outcomes and treatments compared to other DHS patients visiting an English ED, we conduct a regression-based analysis. Our main empirical specification reads as follows:

$$y_{ihmd} = \beta \times Student_i + \gamma \times Ind_i + \omega \times Att_{imd} + \delta\, Severity_{ihmd} + HDFE_{hmd} + \epsilon_{ihmd} \quad (1)$$

In equation (1), y_{ihmd} is the outcome observed for patient i visiting ED h on day d of month m. The vector Ind_i includes individual patient characteristics (age, ethnicity, whether lives in a rural area, income deprivation quintile of the LSOA of residence). The vector Att_{imd} includes characteristics of the patient in relation to the ED attendance, namely: mode of arrival, referral source, whether it is a repeated DSH episode, whether the attendance happens in-hours (between 6AM and 6PM) or out-of-hours (i.e. between 6PM and 6AM, during weekends or bank holidays [31]), and the volume of ED attendances on the same day. The volume of ED attendances on the same day accounts for differences in the level of pressure faced by ED staff, which may influence their behaviour towards patients. The primary ED diagnosis is used as a proxy for severity of DSH episode ($Severity_{ihmd}$). This concurs with ambulance arrivals to account for overall patient severity, which may impact how the DSH patient is treated in the ED. The list and frequencies of ED diagnoses is reported in Appendix 2. The vector HDFE represents a high-dimensional fixed effect (HDFE) that includes ED-specific combinations of month of year and day of week, in relation to the day of the ED attendance. The inclusion of a HDFE allows us to account for various supply and demand side observable and unobservable differences across EDs, and days of attendance, that may affect how DSH patients visiting an ED are treated [31]. In absence of more detailed information of staffing at the time of the attendance, this allows to account for shifts in EDs, that are typically arranged well in advance to accommodate known weekly and seasonal fluctuations in ED demand. Similarly, different EDs may have varied organisational structures and staff-skill mix; that is whether the staff are more or less at ease with patients visiting the department for an episode or DSH. Additionally, on the demand side, university students may be more likely to visits EDs for episodes of DSH at a specific time of year (e.g. during end of term exam periods) or location (e.g. hospitals located in major University cities).

Our main coefficient of interest is β associated with the exposure variable $Student_i$. This reflects the difference in outcomes attributable to being a student according to our proxy definition. In order to interpret β as an unbiased measure of our association of interest, we follow a standard selection on observables argument [32], assuming conditional mean independence. In other words, conditional on the set of characteristics and the high-dimensional fixed effects we control for, the error term ϵ_{ihmd} is mean independent of our student dummy.

For peer review only - http://bmjopen.bmj.com/site/about/guidelines.xhtml

We estimate our models with two related approaches. Firstly, for all outcomes we report estimates of naïve models. These explain outcomes only with our exposure using a Probit model for binary outcomes and a Poisson model for right-skewed outcomes. We then compare our naïve estimates to results from our preferred empirical specification, with and without the more detailed controls for patient severity, represented by primary ED diagnosis. For binary models we assume a linear probability model and estimate equation (1) using a feasible and efficient linear estimator, which accommodates high-dimensional fixed effects implemented in the Stata command *reghdfe* [33]. To account for the skewed distribution of waiting times and the number of investigations and procedures, we estimate models for these outcomes using a Poisson regression with a similar feasible efficient estimator designed to accommodate high-dimensional fixed effects implemented in the Stata command *ppmlhdfe* [34]. For Poisson models we report results as average marginal effects [35].

Sensitivity analyses

To further explore the relationship between our student proxy and outcomes in ED for DSH attendances we propose two stratified analyses. Firstly, we estimate separately models for attendances during (a) standard working hours and (b) out-of-hours respectively, as these periods may differ in the level of ED preparedness and resources. Secondly, we estimate two sets of models for (c) patients brought in by ambulance and (d) arrived at the ED by other means. The rationale for this is that patients arriving by ambulance are likely to have a higher priority. Finally, we estimate the models separately for patients visiting for a first or a repeated DSH episode during our study period. Unfortunately, we are not able to identify repeated DSH episodes before April 2017 due to data limitations. We estimate these sensitivity analyses using our preferred specification including control variables. We are unable to also control for primary ED diagnosis due to the emergence of singleton observations when partialling out the high-dimensional fixed when limiting sample size by splitting observations in sub-groups.

Patient and public involvement

This research did not involve members of the public or patients at any stage, beginning with developing the research questions to identifying the study design or outcomes. We did not consult members of the public in the interpretation of results, nor writing or editing the paper. We also have no specific plans to involve the public in the dissemination of the research findings. Nevertheless, Catherine Campbell developed this research whilst being enrolled as undergraduate student, i.e. as a member of the main target group of this study.

For peer review only - http://bmjopen.bmj.com/site/about/guidelines.xhtml

Results

Seasonality of deliberate self-harm ED attendances

As mentioned in the methods section, there may be seasonality in the attendance of EDs for deliberate self-harm patients. Figure 1 below shows the seasonal variability in the share of patients attending EDs for an episode of deliberate self-harm (averaged across the 127 EDs considered in our analysis), for patients identified as students and non-students.

FIGURE 1 ABOUT HERE

The data suggest a that approximately 1.1 to 1.4 percent of all monthly ED attendances are related to DSH episodes. For patients identified as students there is a clearer seasonality, with higher peaks in October and January, and lower prevalence compared to non-students during the summer months.

Descriptive statistics

Table 1 below shows characteristics of individuals in the sample, based on our main exposure. Patients aged 18-23 identified as students were more likely to be female (73.03 percent compared to 63.00 percent for non-students) and less likely to have a White ethnic background (86.52 percent compared to 93.87 percent for non-students). Students were also less likely to live in rural and deprived areas. There are no substantial differences between students and non-students across the other covariates.

In Table 2 we report summary statistics of outcomes by exposure status, with differences and corresponding test for difference in means and proportions. On average, patients aged 18-23 identified as students wait about 3.03 minutes longer for their initial assessment and receive 0.3 less investigations compared to non-students. They also seem to be less likely to be referred to another provider, to be admitted to hospital, and to have a follow-up visit scheduled at discharge. However, these descriptive differences may be confounded by various observed and unobserved differences between these two groups of young DSH patients.

For peer review only

Table 1: Descriptive statistics for attendances for the full sample and by exposure status

	Full sample		Not flagged as students		Flagged as students	
Variable	Perc.	Count	Perc.	Count	Perc.	Count
Female	64.65	9,099	64.00	8,357	73.03	742
Male	35.35	4,975	36.00	4,701	26.97	274
Ethnicity						
Any White background	93.34	13,136	93.87	12,257	86.52	879
Any Black background	1.45	204	1.33	174	2.95	30
Any Indian background	2.24	315	2.04	266	4.82	49
Any mixed background	2.98	419	2.76	361	5.71	58
Living in rural area	3.20	450	3.36	439	1.08	11
IMD (income component) deprivation quintile						
Least deprived	12.47	1,755	11.26	1,470	28.05	285
2nd quintile	14.22	2,002	14.28	1,865	13.48	137
3rd quintile	19.19	2,701	19.02	2,484	21.36	216
4th quintile	23.58	3,319	23.66	3,089	22.64	230
Most deprived	30.53	4,297	31.78	4,150	14.47	147
ED arrival mode						
Ambulance	48.98	6,893	49.14	6,417	46.85	476
Other	50.67	7,131	50.48	6,592	53.05	539
Not known	0.36	50	0.38	49	0.10	1
Referral source						
Self-referred	51.09	7,191	51.01	6,661	52.17	530
GP	1.58	222	1.53	200	2,17	22
Emergency services	31.04	4,368	31.38	4,097	26.67	271
Health care providers	8.50	1,196	8.29	1,083	11.12	113
Work or education	0.48	68	0.47	62	0.59	6
Other	7.31	1,029	7.31	955	7.28	74
Repeated DSH episodes	35.00	4,926	35.35	4,616	30.51	310
Out of hours (6AM-6PM) attendance	58.18	8,188	57.92	5,495	61.52	625
	Mean (SD)	Range	Mean (SD)	Range	Mean (SD)	Range
Daily ED attendances	416.88 (184.94)	88-1497	412.44 (184.16)	88-1497	474.00 (185.61)	145-1350
Age	20.28 (1.71)	18-23	20.26 (1.72)	18-23	20.43 (1.51)	18-23
Observations	14,074		13,058		1,016	

Notes: (1) The table shows percentages and counts for categorical or binary outcomes, and mean/SD/range for discrete variables at the bottom. (2) The summary statistics refer to the full sample of DSH patients aged 18-23 (first column), and to the sub-samples identified as non-students (second column) and students (third column) based on our student proxy described in the "Methods" section.

Table 2: Summary of outcomes by exposure status

	Not flagged as students				Flagged as students				Difference	
Discrete outcomes	N	Mean	SD	Range	N	Mean	SD	Range	Diff.	*t*
Time to initial assessment	13,058	18.94	21.74	0-80	1,016	21.97	22.74	0-80	3.03***	4.42
Number of investigations	13,058	3.19	3.38	0-12	1,016	2.89	3.40	0-12	-0.29**	-2.67
Binary outcomes	N	Perc.	Count		N	Perc.	Count		Diff.	*z*
Refused or left before treatment	13,058	6.03	787		1,016	5.81	59		-0.22	-0.28
Referred to another provider	12,509	42.06	5,137		978	34.25	335		-6.81***	-4.17
Admitted to hospital	13,058	31.92	4,168		1,016	26.28	267		-5.64***	-3.73
Discharged with no follow-up	12,914	35.27	4,555		1,010	41.09	415		5.82***	3.72
Unplanned follow-up within 7 days	13,058	6.46	844		1,016	4.92	50		-1.54	-1.94

For peer review only

Notes: (1) The table shows percentages and counts for categorical or binary outcomes, and mean/SD/range for discrete outcomes at the top. (2) third column includes the difference between students and non-students, alongside the corresponding t statistic testing the difference in means or z statistic testing the difference in proportions. section. Stars indicate statistical significance as follows: * $p < 0.05$, ** $p < 0.01$, *** $p < 0.001$

For peer review only

Main Results

Table 3 presents the estimated difference attributable to being a student for several indicators of ED performance, compared to non-students. We find a sizeable difference in the number of investigations delivered of -0.262 [-0.491,-0.0327] during the visit (about 8 percent of the baseline average number of investigations for non-students). This difference is consistent across the three empirical specification used (naïve model in column 1, adding patient characteristics and daily volume of attendances at the ED in column 2, and further accounting for differences in severity by controlling for the primary ED diagnosis in column 3). Our multivariate regression analysis suggests that all other differences emerging in the descriptive comparison in Table 2 are likely confounded by other factors.

Table 3: Main results on full sample

		(1)	(2)	(3)
Time to initial assessment	*Estimate*	3.032	-0.958	-1.033
	95% CI	[-1.207,7.271]	[-2.050,0.133]	[-2.130,0.0631]
	N	14074	11581	11579
Number of investigations	*Estimate*	-0.295	-0.402**	-0.262*
	95% CI	[-0.706,0.116]	[-0.680,-0.125]	[-0.491,-0.0327]
	N	14074	11294	11289
Refused or left before treatment	*Estimate*	-0.00220	-0.0101	-0.00914
	95% CI	[-0.0211,0.0167]	[-0.0308,0.0106]	[-0.0262,0.00790]
	N	14074	11852	11852
Referred to another provider	*Estimate*	-0.0681	-0.0273	-0.0107
	95% CI	[-0.143,0.00699]	[-0.0621,0.00753]	[-0.0529,0.0315]
	N	13487	11261	11261
Admitted to hospital	*Estimate*	-0.0564	-0.0235	-0.00143
	95% CI	[-0.118,0.00544]	[-0.0610,0.0140]	[-0.0437,0.0408]
	N	14074	11852	11852
Discharged with no follow-up	*Estimate*	0.0582	0.00601	-0.000976
	95% CI	[-0.0522,0.169]	[-0.0307,0.0427]	[-0.0408,0.0388]
	N	13924	11715	11715
Unplanned follow-up within 7 days	*Estimate*	-0.0154	-0.0204	-0.0221
	95% CI	[-0.0367,0.00584]	[-0.0440,0.00310]	[-0.0456,0.00145]
	N	14074	11852	11852
Controls		No	Patient characteristics<br>Daily att. volume at ED	Patient characteristics<br>Daily att. volume at ED<br>Primary ED diagnosis

Notes: (a) All models and 3 include the following covariates: gender (female or male), age, ethnicity, whether lives in a rural area, income deprivation quintile of the LSOA of residence, mode of arrival, referral source, whether it is a repeated DSH episode, whether the attendance happens in-hours (between 6AM and 6PM) or out-of-hours (between 6PM and 6AM), the volume of ED attendances on the same day at the same ED, primary ED diagnosis. (b) Models were estimated with a Poisson feasible efficient estimator designed to accommodate high-dimensional fixed effects (Correia et al., 2020) for time to initial assessment and number of investigations. For all other outcomes we estimated a linear probability model using a similar feasible efficient linear estimator accommodating high-dimensional fixed effects (Correia, 2016); (c) Estimates represent average marginal effects for the difference between being flagged as students and non-student, 95% confidence intervals in parentheses for standard errors clustered at the ED level. Stars indicate statistical significance as follows: * $p < 0.05$, ** $p < 0.01$, *** $p < 0.001$

For peer review only - http://bmjopen.bmj.com/site/about/guidelines.xhtml

Table 4: Stratified analyses

		In-hours	Out-of-hours	Arrived by ambulance	Did not arrive by ambulance	First DSH episode	Repeated DSH episode
		(1)	(2)	(3)	(4)	(5)	(6)
Time to initial assessment	*Estimate*	-1.511	-0.820	-1.997*	1.611	-1.007	-1.653
	95% CI	[-3.875,0.852]	[-2.339,0.699]	[-3.703,-0.292]	[-1.684,4.905]	[-2.754,0.739]	[-4.206,0.900]
	N	3648	5758	4436	4734	6556	3013
Number of investigations	*Estimate*	-0.387	-0.485**	-0.545***	-0.474*	-0.259	-0.881**
	95% CI	[-0.804,0.0295]	[-0.850,-0.120]	[-0.839,-0.250]	[-0.844,-0.104]	[-0.577,0.0601]	[-1.510,-0.252]
	N	3581	5548	4533	4536	6386	2876
Refused or left before treatment	*Estimate*	-0.00461	-0.0157	-0.0166	-0.00294	-0.0111	-0.00441
	95% CI	[-0.0341,0.0248]	[-0.0433,0.0119]	[-0.0468,0.0135]	[-0.0298,0.0239]	[-0.0412,0.0191]	[-0.0360,0.0272]
	N	3779	5910	4728	4973	6733	3125
Referred to another provider	*Estimate*	-0.0334	-0.0140	-0.0708*	-0.0230	-0.0202	-0.0539
	95% CI	[-0.109,0.0425]	[-0.0585,0.0306]	[-0.140,-0.00182]	[-0.0837,0.0377]	[-0.0633,0.0229]	[-0.169,0.0614]
	N	3553	5603	4459	4707	6402	2922
Admitted to hospital	*Estimate*	-0.00424	-0.00631	-0.0602	-0.0119	-0.0195	0.00206
	95% CI	[-0.0762,0.0678]	[-0.0476,0.0350]	[-0.127,0.00680]	[-0.0760,0.0522]	[-0.0534,0.0144]	[-0.0891,0.0932]
	N	3779	5910	4728	4973	6733	3125
Discharged with no follow-up	*Estimate*	-0.00424	0.00450	0.0276	-0.0122	0.0260	-0.0336
	95% CI	[-0.0762,0.0678]	[-0.0442,0.0532]	[-0.0258,0.0811]	[-0.0641,0.0397]	[-0.0156,0.0675]	[-0.117,0.0494]
	N	3779	5829	4681	4885	6661	3070
Unplanned follow-up within 7 days	*Estimate*	-0.00782	-0.0306*	-0.0182	-0.0211	-0.0179	-0.0442
	95% CI	[-0.0605,0.0449]	[-0.0576,-0.00363]	[-0.0463,0.00997]	[-0.0533,0.0110]	[-0.0556,0.0199]	[-0.146,0.0573]
	N	3728	5910	4728	4973	6733	3125

Notes: (a) All models and 3 include the following covariates: gender (female or male), age, ethnicity, whether lives in a rural area, income deprivation quintile of the LSOA of residence, mode of arrival, referral source, whether it is a repeated DSH episode, whether the attendance happens in-hours (between 6AM and 6PM) or out-of-hours (between 6PM and 6AM), the volume of ED attendances on the same day at the same ED. (b) Models were estimated with a Poisson feasible efficient estimator designed to accommodate high-dimensional fixed effects (Correia et al., 2020) for time to initial assessment and number of investigations. For all other outcomes we estimated a linear probability model using a similar feasible efficient linear estimator accommodating high-dimensional fixed effects (Correia, 2016); (c) Estimates represent average marginal effects for the difference between being flagged as students and non-student, 95% confidence intervals in parentheses for standard errors clustered at the ED level. Stars indicate statistical significance as follows: * $p < 0.05$, ** $p < 0.01$, *** $p < 0.001$

For peer review only

Sensitivity Analysis

In Table 4 we report estimates for our specification including control variables (but not primary ED diagnosis) for our three sensitivity checks, namely the stratified analyses by arrival time (in-hours vs. out-of-hours), arrival mode (patients brought in by ambulance vs. arriving at the ED by other means) and first or repeated DSH episode. Our main finding of fewer investigations delivered to patients identified as students seems concentrated amongst out-of-hours visits (column 2) and patients visiting for repeated DSH episodes (column 6, as opposed to the first DSH episode in the study period in column 5). Patient visiting during nights, weekends or bank holidays are also less likely to have an unplanned follow-up visit within 7 days (-0.0306 [-0.0576,-0.00363]).

Columns 3 and 4 reflect the differences in outcomes attributable to whether the patient arrived by ambulance, comparing students and non-students. Whilst the main results in Table 3 account for the mode of arrival, splitting DSH patients by ambulance arrivals may help to better account for an intrinsic unobserved heterogeneity between these patient groups. We find that students who arrive by ambulance wait about two less minutes for initial assessment (-1.997 [-3.703,-0.292]) and are about 7 percent less likely (-0.0708 [-0.140,-0.00182]) to be referred to another provider. The number of investigations performed at a patient's initial assessment for both arrival modes is consistent with our main results.

Discussion

Summary of findings

Our analysis proposes a method to identify patients who are likely students in the HES ED records, and explores differences in healthcare outcomes between students and non-students attending an ED after an episode of deliberate self-harm. We find a statistically significant difference of 0.262 less investigations delivered to students compared to non-students, which is equivalent to about 8 percent compared to the baseline number of investigations for non-students. All other differences in ED care outcomes are not statistically significant in our main analysis. The results of our stratified analyses by time of attendance (regular times compared to out of hours, i.e. 6PM to 6AM and weekends), ambulance arrival, and first vs. repeated DSH episode confirm the main findings providing a more nuanced picture. The reduced number of investigations (compared to non-students) is concentrated amongst students visiting EDs during out-of-

For peer review only - http://bmjopen.bmj.com/site/about/guidelines.xhtml

For peer review only

hours and for repeated DSH episodes. Students visiting during out-of-hours are also less likely to have unplanned follow-up visits within 7 days. On the other hand, students arriving at the ED by ambulance seem to wait slightly less for initial assessment and be less likely to be referred to another provider.

Strengths and weaknesses of the study

This paper has several strengths. Firstly, our analysis uses a large dataset capturing ED attendances of DSH patients in English hospitals for an entire year. Secondly, it trials a student proxy indicator to identify and measure differences in emergency care outcomes compared to other patients attending EDs for the same reason – deliberate self-harm. Thirdly, in order to isolate the association between ED care outcomes and being a student, we address potential sources of confounding both on the demand (i.e. patients) and supply (i.e. hospital) sides. To account for compositional differences and unobserved patient severity, our main specification controls for patient characteristics and primary ED diagnosis. On the supply side, existing literature finds that – based on historical attendance patterns – some variation in attendance volume is predictable and as a result EDs are capable of adjusting capacity accordingly [36]. To circumvent the confounding effect on estimates resulting from ED shift scheduling patterns and differing times of attendance, we control for number of ED attendances on the same day and include an ED-specific high-dimensional fixed effect to clean our estimates from expected patterns of shift planning and natural variations in ED attendance across areas and seasons.

Despite our range of sensitivity checks, some limitations remain. First, our student indicator is only a proxy, and therefore, be interpreted with caution. The marked difference in seasonality of DSH episodes (Figure 1) and the geographical location of students' areas of residence (Appendix 1) provide some validation. Nevertheless, our method of identifying students is conservative, suggesting our results are likely interpretable as lower bounds of student attendance volumes and related differences in ED care. Moreover, our study does not include any qualitative component; we do not interview ED staff members in regards to their attitude towards self-harm patients, a practice which would help to interpret some of our findings [12]. Finally, variability in the systems and protocol to assess and treat self-harm presentations may impede a meaningful comparison across areas [37]. We try to circumvent this latter aspect by accounting for hospital-specific fixed effects, and by exploring the heterogeneity in the results across areas more or less exposed to, and equipped for, episodes of deliberate self-harm.

For peer review only - http://bmjopen.bmj.com/site/about/guidelines.xhtml

Strengths and weaknesses in relation to other studies

A related study by Chartrand et al. [38] proposes a longitudinal analysis of differences in discharge destination and rate of reattendance between patients who experience DSH and those who attempt suicide. They find those who re-attended for DSH and suicidal attempt were more likely to be discharged to usual care during the initial assessment compared to a patient only attending for DSH. In view of this, a potential weakness of our study – dictated by data limitations – is that we did not include an indicator for suicide attempts. This is an important factor as patients who engage in DSH are at greater risk of subsequently dying by suicide [16]. Our results were consistent with a paper by Sinclair et al. [39] whom employs a crossover methodological design to examine the differences in care before and after the introduction of a dedicated psychiatric nurse service. Importantly, the author finds an increase in the accuracy of assessment and diagnosis post-intervention, wherein nurses are better trained to address DSH patients during initial assessments. These results may be consistent with a higher effectiveness in assessing patients, and hence explain the presented negative association between patient characteristics (being a student attending for DSH) and the number of investigations.

University students are not a homogenous group; rather there is large variation in health-seeking behaviours within the student population and compared to other patients in the same age group. Existing literature find that upon arrival at the ED, students experience feelings of shame and fear surrounding a perceived 'failure to cope' and lack of confidentiality [40,41]. It is plausible that these feelings are exacerbated when attending outside of normal working hours, pushing these patients to form a preference for not reattending the ED in case of need [42].

The decreased number of investigations amongst students, compared to non-students, is consistent with physician's actions being guided by a both a priority to treat physical symptoms (over psychological symptoms) and limited knowledge in terms of the immediate management of self-harm patients. In particular, qualitative literature finds self-harm patients experience negative attitudes from staff and differences in treatment compared to other patients [43]. These patterns may originate from an increased level of uncertainty in dealing with this patient group, wherein a deficit in self-harm training has be emphasised [44]. Previous literature has also raised concerns over a lack of local policies that prioritise mental health over physical health [45]. Finally, the larger difference in the number of investigations for students visiting EDs for repeated deliberate self-harm episodes may reflect either a better specific

For peer review only

knowledge of the specific patient by ED staff or a marker of a generalised attitude toward students frequently attending EDs for mental health related problems materialising in episodes of self-harm [12,13].

In addition, the reduced number of investigations recorded amongst students compared to non-students irrespective of the arrival mode (i.e. ambulance or not) may reflect faults in the ED triage process. The first step upon arrival requires patients to provide a brief summary of their health condition(s) at reception. Following this, patients wait for an initial assessment, wherein a medical or nursing professional determines priority for treatment by taking a brief medical history, a pain assessment and any collection of early warning scores [46]. Therefore, if staff are not trained in the management of mental health patients, EDs overlook a crucial opportunity to diagnose mental illness and commence a treatment pathway that may prevent subsequent self-harming behaviour [47].

Policy Implications

Poor mental health is a major clinical priority given that it is at least partially preventable and a contributing risk factor for suicide attempts, especially among teens and young adults [15]. Our results have implications for both university health care and ED systems. English universities need to further invest in resources to address the heightened demands for counselling services [48]. Oversubscription and a shortage of trained staff have impacted services through reduced length and number of sessions, longer waitlists, and more referrals to off-campus providers [2]. In addition, university health centres need to prioritise information campaigns to decrease the stigma surrounding self-harm that prevents many students from seeking help, and consequently, lead to EDs being a first point of contact. The higher probability of refusing or leaving without treatment when attending EDs in-hours suggests the need for greater access to dedicated mental health staff or treatment, better access to community-based resources, increased availability of inpatient psychiatric beds and a separate safe space in the ED [49]. In addition, roughly one half of DSH patients in ED do not receive a psychosocial assessment [50], which further indicates the need to regulate and standardise mental health services. This suggest that better coordination between university mental health services and EDs may be needed in the long-term. In the short-term, in view of existing time and resource constraints, educating existing staff in the evaluation and management of DSH patients may be the only feasible approach to address such issues.

Open questions and future research

For peer review only

This paper could be advanced in several ways. Further research is needed to investigate the association between students and ED outcomes amongst those who have current contact with mental health services at the time of self-harm. Future studies should aim to understand differences in student's health-seeking behaviour that could help design policies to mitigate staff bias towards this sub-group. Future research should also focus on the scarcity of integrated inpatient and outpatient mental health services at the county level. This is crucial in providing unified health care for a high-risk and vulnerable population, whilst also reducing capacity constraints on emergency departments.

For peer review only

Figure captions

Figure 1: Share of DSH patients of all ED attendances by month

Note: The figure shows the overall share of DSH attendances in each month across all 127 EDs in our sample, split between patients aged 18-23 identified as students and non-students using the proxy outlined in the "Methods" section.

Declarations

Authors' contributions: CC and IF developed the original idea for the study, designed the analysis and carried out the analysis. CC, IF and JD contributed substantially to the interpretation of results and the writing of the manuscript. IF is responsible for the overall content as the guarantor.

Funding: This research was supported by the National Institute for Health Research (NIHR) Applied Research Collaboration Greater Manchester (NIHR200174).

Competing interests: None to declare

Data availability statement: The Hospital Episode Statistics data that support the findings of this study are available from NHS Digital. Restrictions apply to the availability of these data. They were used under licence for this study, and so are not publicly available.

Patient consent for publication: Not applicable.

Ethics approval: Not applicable.

Acknowledgments: We are grateful for comments and suggestions received from colleagues during Health Organisation, Policy and Economics (HOPE) seminars at the University of Manchester. Additional thanks to the Winter 2022 Health Economics Study Group (HESG) conference participants in Leeds for feedback during the poster session.

For peer review only

References

1 Kessler RC, Amminger GP, Aguilar-Gaxiola S, *et al.* Age of onset of mental disorders: a review of recent literature. *Curr Opin Psychiatry*. 2007;20:359–64.

2 The WHO World Mental Health Survey Consortium. Prevalence, Severity, and Unmet Need for Treatment of Mental Disorders in the World Health Organization World Mental Health Surveys. *JAMA*. 2004;291:2581–90.

3 Dodd AL. Student mental health research: moving forwards with clear definitions. *J Ment Health*. 2021;30:273–5.

4 Storrie K, Ahern K, Tuckett A. A systematic review: Students with mental health problems—A growing problem. *Int J Nurs Pract*. 2010;16:1–6.

5 Gallagher RP. Thirty Years of the National Survey of Counseling Center Directors: A Personal Account. *J Coll Stud Psychother*. 2012;26:172–84.

6 Gunnell D, Caul S, Appleby L, *et al.* The incidence of suicide in University students in England and Wales 2000/2001–2016/2017: Record linkage study. *J Affect Disord*. 2020;261:113–20.

7 Macaskill A. The mental health of university students in the United Kingdom. *Br J Guid Couns*. 2013;41:426–41.

8 McManus S, Gunnell D. Trends in mental health, non-suicidal self-harm and suicide attempts in 16–24-year old students and non-students in England, 2000–2014. *Soc Psychiatry Psychiatr Epidemiol*. 2020;55:125–8.

9 Querstret D. Collaborating with Students to Support Student Mental Health and Well-being. In: Lygo-Baker S, Kinchin IM, Winstone NE, eds. *Engaging Student Voices in Higher Education : Diverse Perspectives and Expectations in Partnership*. Cham: Springer International Publishing 2019:191–207. https://doi.org/10.1007/978-3-030-20824-0_12

10 Wilson C, Spencer G. The mental health support needs of university students with long-term physical health conditions. *Health Educ*. 2022;122:584–98.

11 Yorgason JB, Roper SO, Sandberg JG, *et al.* Stress spillover of health symptoms from healthy spouses to patient spouses in older married couples managing both diabetes and osteoarthritis. *Fam Syst Health*. 2012;30:330–43.

12 Mackay N, Barrowclough C. Accident and emergency staff's perceptions of deliberate self-harm: Attributions, emotions and willingness to help. *Br J Clin Psychol*. 2005;44:255–67.

13 Cleaver K. Attitudes of emergency care staff towards young people who self-harm: A scoping review. *Int Emerg Nurs*. 2014;22:52–61.

14 Timson D, Priest H, Clark-Carter D. Adolescents who self-harm: Professional staff knowledge, attitudes and training needs. *J Adolesc*. 2012;35:1307–14.

15 Beckman K, Mittendorfer-Rutz E, Waern M, *et al.* Method of self-harm in adolescents and young adults and risk of subsequent suicide. *J Child Psychol Psychiatry*. 2018;59:948–56.

16 Bergen H, Hawton K, Waters K, *et al.* How do methods of non-fatal self-harm relate to eventual suicide? *J Affect Disord*. 2012;136:526–33.

17 Chan MKY, Bhatti H, Meader N, *et al.* Predicting suicide following self-harm: systematic review of risk factors and risk scales. *Br J Psychiatry*. 2016;209:277–83.

18 Hawton K, Bergen H, Kapur N, *et al.* Repetition of self-harm and suicide following self-harm in children and adolescents: findings from the Multicentre Study of Self-harm in England. *J Child Psychol Psychiatry*. 2012;53:1212–9.

19 Bolotnyy V, Basilico M, Barreira P. Graduate Student Mental Health: Lessons from American Economics Departments. *J Econ Lit*. 2022;60:1188–222.

20 Macchi E, Sievert C, Bolotnyy V, *et al.* Mental Health in European Economics Departments. 2023. https://doi.org/10.2139/ssrn.4509725

21 Eisenberg D, Golberstein E, Gollust SE. Help-Seeking and Access to Mental Health Care in a University Student Population. *Med Care*. 2007;45:594–601.

22 Ziano I, Koc Y. Prototypes of People With Depression. *Psychol Sci*. 2023;34:1295–308.

23 Acampora M, Capozza F, Moghani V. Mental Health Literacy, Beliefs and Demand for Mental Health Support. 2022. https://doi.org/10.2139/ssrn.4261487

For peer review only

24 Holt M, Powell S. Healthy Universities: a guiding framework for universities to examine the distinctive health needs of its own student population. *Perspect Public Health*. 2017;137:53–8.

25 Tran D-MT, Silvestri-Elmore A. Healthcare-seeking behaviours in college students and young adults: a review. *J Res Nurs*. 2021;26:320–38.

26 NHS England. Hospital Episode Statistics (HES). NHS Digit. 2023. https://digital.nhs.uk/data-and-information/data-tools-and-services/data-services/hospital-episode-statistics (accessed 10 August 2023)

27 Department for Communities and Local Government. English indices of deprivation 2015. 2015. https://www.gov.uk/government/statistics/english-indices-of-deprivation-2015

28 The King's Fund. What's going on with A&E waiting times? 2020. https://www.kingsfund.org.uk/projects/urgent-emergency-care/urgent-and-emergency-care-mythbusters (accessed 11 May 2022)

29 NHS Digital. Patients Registered at a GP Practice. NHS Digit. 2022. https://digital.nhs.uk/data-and-information/publications/statistical/patients-registered-at-a-gp-practice (accessed 19 August 2022)

30 Anselmi L, Everton A, Shaw R, *et al.* Estimating local need for mental healthcare to inform fair resource allocation in the NHS in England: cross-sectional analysis of national administrative data linked at person level. *Br J Psychiatry*. 2020;216:338–44.

31 Turner AJ, Anselmi L, Lau Y-S, *et al.* The effects of unexpected changes in demand on the performance of emergency departments. *Health Econ*. 2020;29:1744–63.

32 Altonji JG, Elder TE, Taber CR. Selection on Observed and Unobserved Variables: Assessing the Effectiveness of Catholic Schools. *J Polit Econ*. 2005;113:151–84.

33 Correia S. A Feasible Estimator for Linear Models with Multi-Way Fixed Effects. 2016. http://scorreia.com/research/hdfe.pd

34 Correia S, Guimarães P, Zylkin T. Fast Poisson estimation with high-dimensional fixed effects. *Stata J*. 2020;20:95–115.

35 Norton EC, Dowd BE, Maciejewski ML. Marginal Effects—Quantifying the Effect of Changes in Risk Factors in Logistic Regression Models. *JAMA*. 2019;321:1304–5.

36 Anselmi L, Meacock R, Kristensen SR, *et al.* Arrival by ambulance explains variation in mortality by time of admission: retrospective study of admissions to hospital following emergency department attendance in England. *BMJ Qual Saf*. 2017;26:613–21.

37 Joyce M, Chakraborty S, O'Sullivan G, *et al.* National Self-Harm Registry Ireland Annual Report 2020. Cork: National Suicide Research Foundation 2022.

38 Chartrand H, Tefft B, Sareen J, *et al.* A Longitudinal Study of Correlates, Discharge Disposition, and Rate of Re-presentation to Emergency Services of Adults who Engage in Non-suicidal Self-injury. *Arch Suicide Res*. 2022;26:1141–58.

39 Sinclair L, Hunter R, Hagen S, *et al.* How effective are mental health nurses in A&E departments? *Emerg Med J*. 2006;23:687–92.

40 Owens C, Hansford L, Sharkey S, *et al.* Needs and fears of young people presenting at accident and emergency department following an act of self-harm: secondary analysis of qualitative data. *Br J Psychiatry*. 2016;208:286–91.

41 Mulhearne P, Cotter P, O'Shea M, *et al.* Experiences of registered general nurses who care for patients presenting with self-harm to the emergency department in Ireland. *Int Emerg Nurs*. 2021;58:101047.

42 Cleaver K, Meerabeau L, Maras P. Attitudes towards young people who self-harm: age, an influencing factor. *J Adv Nurs*. 2014;70:2884–96.

43 Gibson R, Carson J, Houghton T. Stigma towards non-suicidal self-harm: evaluating a brief educational intervention. *Br J Nurs*. 2019;28:307–12.

44 Wolf LA, Perhats C, Delao AM. US emergency nurses' perceptions of challenges and facilitators in the management of behavioural health patients in the emergency department: A mixed-methods study. *Australas Emerg Nurs J*. 2015;18:138–48.

45 Department of Health. A&E clinical quality indicators: Implementation guidance and data definitions. 2010. https://webarchive.nationalarchives.gov.uk/ukgwa/20130105030902mp_/http://www.dh.gov.uk/en/

For peer review only

Publicationsandstatistics/Publications/PublicationsPolicyAndGuidance/DH_122868 (accessed 5 January 2024)

46 Butler C, Joiner R, Bradley R, *et al.* Self-harm prevalence and ideation in a community sample of cis, trans and other youth. *Int J Transgenderism*. 2019;20:447–58.

47 Furber G, Segal L, Leach M, *et al.* Preventing mental illness: closing the evidence-practice gap through workforce and services planning. *BMC Health Serv Res*. 2015;15:283.

48 Prince JP. University student counseling and mental health in the United States: Trends and challenges. *Ment Health Prev*. 2015;3:5–10.

49 Cullen SW, Diana A, Olfson M, *et al.* If You Could Change 1 Thing to Improve the Quality of Emergency Care for Deliberate Self-harm Patients, What Would It Be? A National Survey of Nursing Leadership. *J Emerg Nurs*. 2019;45:661–9.

50 Bennewith O, Peters TJ, Hawton K, *et al.* Factors associated with the non-assessment of self-harm patients attending an Accident and Emergency Department: Results of a national study. *J Affect Disord*. 2005;89:91–7.

For peer review only

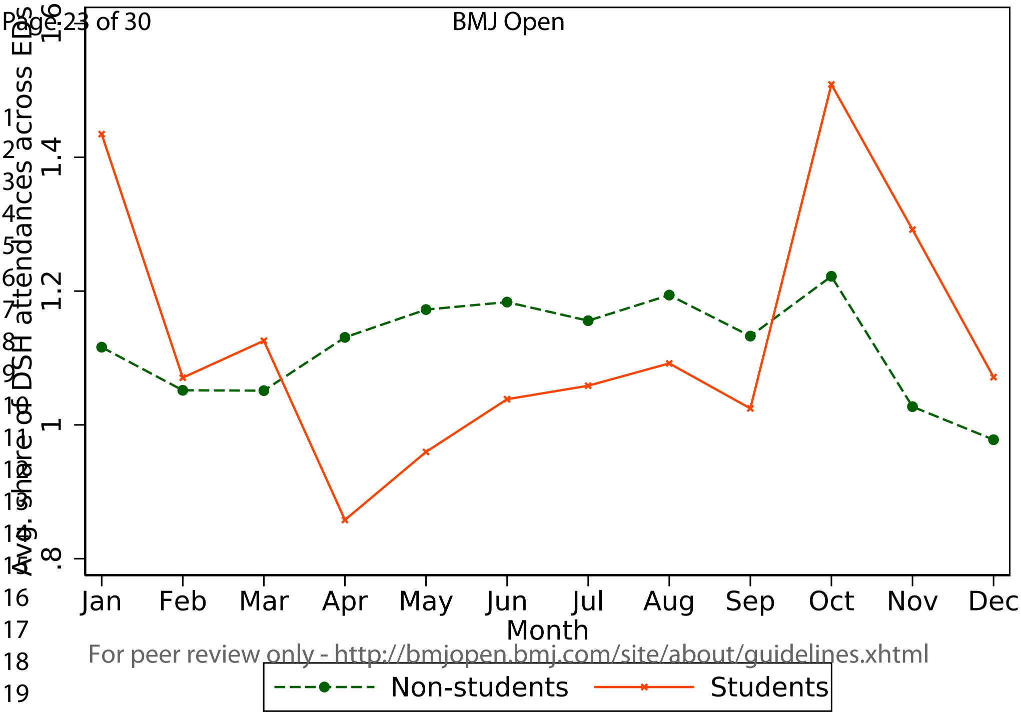
Avg. share of DSH attendances across EDs
1.6
1.4
1.2
1
.8
Jan
Feb
Mar
Apr
May
Jun
Jul
Aug
Sep
Oct
Nov
Dec
Month
Non-students
Students

Supplementary material to the paper titled "Outcomes for university students following emergency care presentation for deliberate self-harm: retrospective observational study of Emergency Departments in England for 2017/2018"

Authors: Catherine Campbell, Joe Dodd, Igor Francetic

Version: January 2024.

Appendix 1: Distribution of LSOAs with more than 10 percent of residents aged 18-23

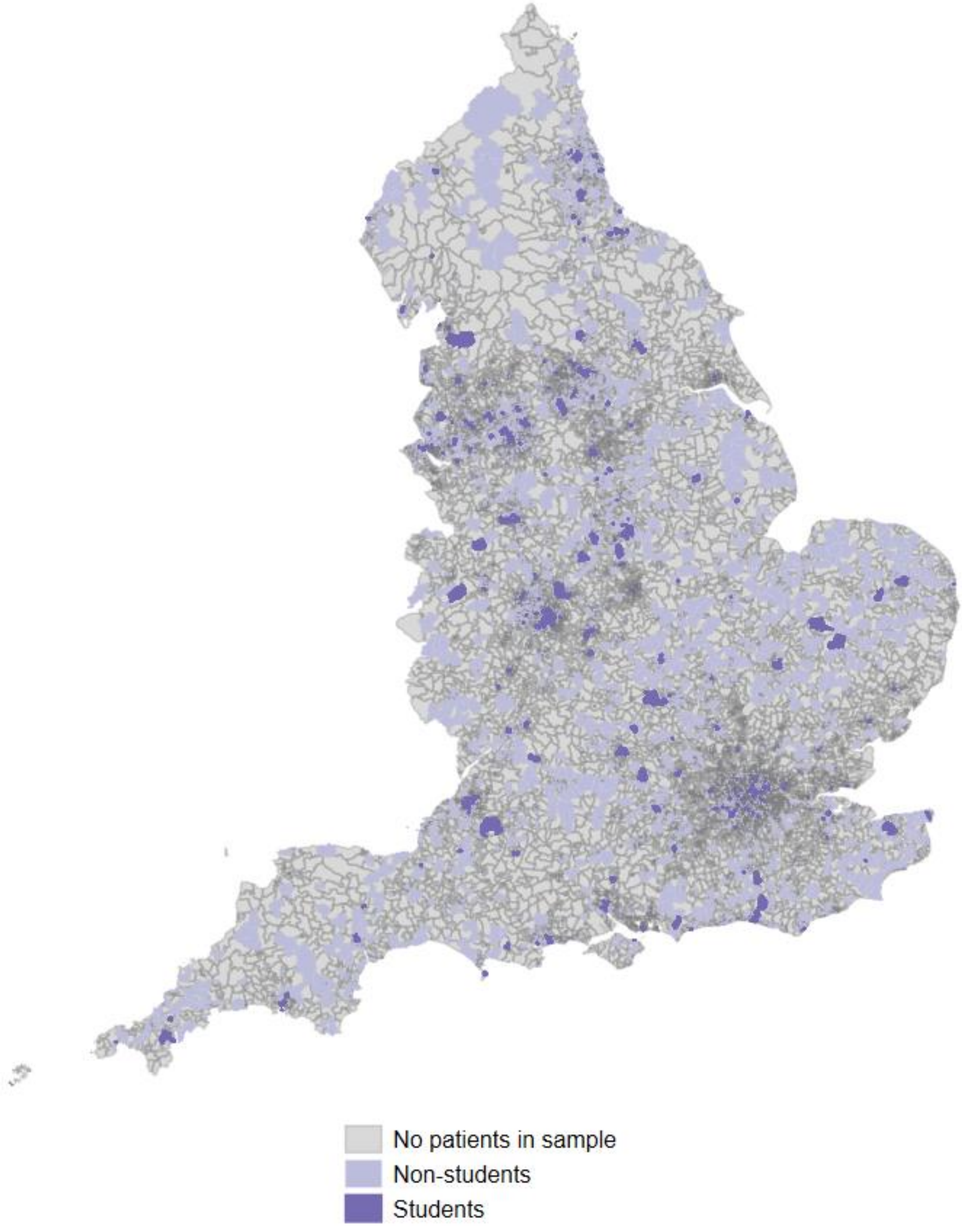

Note: Grey areas represent LSOAs in England that are not represented in our sample. Areas in light purple represent LSOAs of residence for patients not identified as students in our sample. Areas in darker purple represent LSOAs of residence of patients identified as students. The latter are clustered around university areas in England, for example Exeter, Bristol, Bath, Reading, London, Oxford, Cambridge, Birmingham, Coventry, Norwich, Liverpool, Manchester, Leeds, Sheffield, York, Lancaster, Durham and Newcastle among others.

Appendix 2: Distribution of primary ED codes (severity proxy) for analytical sample of attendances

Primary ED Diagnosis	Freq.	Percent
Laceration	1,244	8.84
Contusion/abrasion	360	2.56
Soft tissue inflammation	95	0.68
Head injury	108	0.77
Dislocation/fracture/joints	281	2.00
Sprain/ligament injury	107	0.76
Muscle/tendon injury	43	0.31
Burns and scalds	33	0.23
Electric shock	1	0.01
Foreign body	227	1.61
Bites/stings	13	0.09
Poisoning (inc overdose)	4,780	33.96
Near drowning	2	0.01
Visceral injury	6	0.04
Infectious disease	3	0.02
Local infection	24	0.17
Septicaemia	1	0.01
Cardiac conditions	28	0.20
Cerebro-vascular conditions	3	0.02
Other vascular conditions	2	0.01
Haematological conditions	4	0.03
Central nervous system conditions	43	0.31
Respiratory conditions	14	0.10
Gastrointestinal conditions	60	0.43
Urological conditions (inc cystitis)	12	0.09
Obstetric conditions	1	0.01
Gynaecological conditions	36	0.26
Diabetes and other endocrinological conditions	15	0.11
Dermatological conditions	6	0.04
Allergy (inc anaphylaxis)	3	0.02
Facio-maxillary conditions	2	0.01
ENT conditions	8	0.06
Psychiatric conditions	2,154	15.30
Ophthalmological conditions	17	0.12
Social problems (inc chronic alcoholism)	94	0.67
Diagnosis not classifiable	1,407	10.00
Nothing abnormal detected	189	1.34
Missclassified	703	5.00
No/missing diagnosis	1,945	13.82
Total	14,074	100

For peer review only

The RECORD statement – checklist of items, extended from the STROBE statement, that should be reported in observational studies using routinely collected health data.

	Item No.	STROBE items	Location in manuscript where items are reported	RECORD items	Location in manuscript where items are reported
**Title and abstract**					
	1	(a) Indicate the study's design with a commonly used term in the title or the abstract (b) Provide in the abstract an informative and balanced summary of what was done and what was found	Title and abstract – page 1.	RECORD 1.1: The type of data used should be specified in the title or abstract. When possible, the name of the databases used should be included.<br><br>RECORD 1.2: If applicable, the geographic region and timeframe within which the study took place should be reported in the title or abstract.<br><br>RECORD 1.3: If linkage between databases was conducted for the study, this should be clearly stated in the title or abstract.	Title and abstract – page 1.
**Introduction**					
Background rationale	2	Explain the scientific background and rationale for the investigation being reported	Introduction – page 1.		
Objectives	3	State specific objectives, including any prespecified hypotheses	Introduction, final paragraph – pages 1 & 2.		
**Methods**					
Study Design	4	Present key elements of study design early in the paper	Methods – pages 2-4.		
Setting	5	Describe the setting, locations, and relevant dates, including periods of recruitment, exposure, follow-up, and data collection	Methods, subsections: 'data and sample' (page 2) and 'exposure' (page 3).		

For peer review only

Participants	6	*(a) Cohort study* - Give the eligibility criteria, and the sources and methods of selection of participants. Describe methods of follow-up<br>*Case-control study* - Give the eligibility criteria, and the sources and methods of case ascertainment and control selection. Give the rationale for the choice of cases and controls<br>*Cross-sectional study* - Give the eligibility criteria, and the sources and methods of selection of participants<br><br>*(b) Cohort study* - For matched studies, give matching criteria and number of exposed and unexposed<br>*Case-control study* - For matched studies, give matching criteria and the number of controls per case	Methods, subsection 'data and sample' – page 2.	RECORD 6.1: The methods of study population selection (such as codes or algorithms used to identify subjects) should be listed in detail. If this is not possible, an explanation should be provided.<br><br>RECORD 6.2: Any validation studies of the codes or algorithms used to select the population should be referenced. If validation was conducted for this study and not published elsewhere, detailed methods and results should be provided.<br><br>RECORD 6.3: If the study involved linkage of databases, consider use of a flow diagram or other graphical display to demonstrate the data linkage process, including the number of individuals with linked data at each stage.	Methods, subsection 'data and sample' – page 2.
Variables	7	Clearly define all outcomes, exposures, predictors, potential confounders, and effect modifiers. Give diagnostic criteria, if applicable.	Methods, subsections 'exposure', 'outcomes' and 'empirical approach' – pages 3 & 4.	RECORD 7.1: A complete list of codes and algorithms used to classify exposures, outcomes, confounders, and effect modifiers should be provided. If these cannot be reported, an explanation should be provided.	Methods, subsection 'empirical approach' – pages 3 & 4.
Data sources/ measurement	8	For each variable of interest, give sources of data and details of methods of assessment (measurement).<br>Describe comparability of assessment methods if there is more than one group	Methods – pages 2-4.		

For peer review only

Bias	9	Describe any efforts to address potential sources of bias	Methods, subsection 'empirical approach' – page 4.		
Study size	10	Explain how the study size was arrived at	Methods, subsections 'data and sample' and 'exposure' – pages 2 & 3.		
Quantitative variables	11	Explain how quantitative variables were handled in the analyses. If applicable, describe which groupings were chosen, and why	Methods, subsection 'empirical approach' – pages 3 & 4.		
Statistical methods	12	(a) Describe all statistical methods, including those used to control for confounding<br>(b) Describe any methods used to examine subgroups and interactions<br>(c) Explain how missing data were addressed<br>(d) *Cohort study* - If applicable, explain how loss to follow-up was addressed<br>*Case-control study* - If applicable, explain how matching of cases and controls was addressed<br>*Cross-sectional study* - If applicable, describe analytical methods taking account of sampling strategy<br>(e) Describe any sensitivity analyses	Methods, subsections 'empirical approach' and 'sensitivity analyses' – pages 3 & 4.		
Data access and cleaning methods		..		RECORD 12.1: Authors should describe the extent to which the investigators had access to the database	Methods, subsection 'data and sample' – page 2.

For peer review only

				population used to create the study population.<br><br>RECORD 12.2: Authors should provide information on the data cleaning methods used in the study.	
Linkage		..		RECORD 12.3: State whether the study included person-level, institutional-level, or other data linkage across two or more databases. The methods of linkage and methods of linkage quality evaluation should be provided.	Methods, subsection 'data and sample' – page 2.
**Results**					
Participants	13	(a) Report the numbers of individuals at each stage of the study (*e.g.*, numbers potentially eligible, examined for eligibility, confirmed eligible, included in the study, completing follow-up, and analysed)<br>(b) Give reasons for non-participation at each stage.<br>(c) Consider use of a flow diagram	Methods, subsections 'data and sample' and 'exposure' – pages 2 & 3.	RECORD 13.1: Describe in detail the selection of the persons included in the study (*i.e.,* study population selection) including filtering based on data quality, data availability and linkage. The selection of included persons can be described in the text and/or by means of the study flow diagram.	Methods, subsections 'data and sample' and 'exposure' – pages 2 & 3.
Descriptive data	14	(a) Give characteristics of study participants (*e.g.*, demographic, clinical, social) and information on exposures and potential confounders<br>(b) Indicate the number of participants with missing data for each variable of interest<br>(c) *Cohort study* - summarise follow-up time (*e.g.*, average and total amount)	Results, subsection 'descriptive statistics' – pages 5 & 6.		

For peer review only

Outcome data	15	*Cohort study* - Report numbers of outcome events or summary measures over time<br>*Case-control study* - Report numbers in each exposure category, or summary measures of exposure<br>*Cross-sectional study* - Report numbers of outcome events or summary measures	Results, subsection 'descriptive statistics' – pages 7.		
Main results	16	(a) Give unadjusted estimates and, if applicable, confounder-adjusted estimates and their precision (e.g., 95% confidence interval). Make clear which confounders were adjusted for and why they were included<br>(b) Report category boundaries when continuous variables were categorized<br>(c) If relevant, consider translating estimates of relative risk into absolute risk for a meaningful time period	Results, subsection 'main results' – pages 7.		
Other analyses	17	Report other analyses done—e.g., analyses of subgroups and interactions, and sensitivity analyses	Results, subsection 'sensitivity analyses' – pages 7 & 8.		
**Discussion**					
Key results	18	Summarise key results with reference to study objectives	Discussion, subsection 'summary of findings' – page 8 & 9.		
Limitations	19	Discuss limitations of the study, taking into account sources of potential bias or imprecision.	Discussion – pages 9 & 10.	RECORD 19.1: Discuss the implications of using data that were not created or collected to answer the specific research question(s). Include	Discussion – pages 9 & 10.

For peer review only

		Discuss both direction and magnitude of any potential bias		discussion of misclassification bias, unmeasured confounding, missing data, and changing eligibility over time, as they pertain to the study being reported.	
Interpretation	20	Give a cautious overall interpretation of results considering objectives, limitations, multiplicity of analyses, results from similar studies, and other relevant evidence	Discussion, pages 8-10.		
Generalisability	21	Discuss the generalisability (external validity) of the study results			
**Other Information**					
Funding	22	Give the source of funding and the role of the funders for the present study and, if applicable, for the original study on which the present article is based	Declarations, page 12.		
Accessibility of protocol, raw data, and programming code		..		RECORD 22.1: Authors should provide information on how to access any supplemental information such as the study protocol, raw data, or programming code.	

*Reference: Benchimol EI, Smeeth L, Guttmann A, Harron K, Moher D, Petersen I, Sørensen HT, von Elm E, Langan SM, the RECORD Working Committee. The REporting of studies Conducted using Observational Routinely-collected health Data (RECORD) Statement. *PLoS Medicine* 2015; in press.

*Checklist is protected under Creative Commons Attribution (CC BY) license.

For peer review only

BMJ Open

Outcomes for university students following emergency care presentation for deliberate self-harm: a retrospective observational study of emergency departments in England for 2017/2018

Journal:	*BMJ Open*
Manuscript ID	bmjopen-2023-078672.R2
Article Type:	Original research
Date Submitted by the Author:	18-Jan-2024
Complete List of Authors:	Campbell, Catherine; The University of Manchester, Centre for Primary Care and Health Services Research<br>Dodd, Joe; The University of Manchester, Centre for Primary Care and Health Services Research<br>Francetic, Igor; The University of Manchester, Centre for Primary Care and Health Services Research
**Primary Subject Heading**:	Emergency medicine
Secondary Subject Heading:	Mental health, Health services research, Public health, Research methods
Keywords:	Suicide & self-harm < PSYCHIATRY, ACCIDENT & EMERGENCY MEDICINE, MENTAL HEALTH, Observational Study

SCHOLARONE™
Manuscripts

I, the Submitting Author has the right to grant and does grant on behalf of all authors of the Work (as defined in the below author licence), an exclusive licence and/or a non-exclusive licence for contributions from authors who are: i) UK Crown employees; ii) where BMJ has agreed a CC-BY licence shall apply, and/or iii) in accordance with the terms applicable for US Federal Government officers or employees acting as part of their official duties; on a worldwide, perpetual, irrevocable, royalty-free basis to BMJ Publishing Group Ltd ("BMJ") its licensees and where the relevant Journal is co-owned by BMJ to the co-owners of the Journal, to publish the Work in this journal and any other BMJ products and to exploit all rights, as set out in our licence.

The Submitting Author accepts and understands that any supply made under these terms is made by BMJ to the Submitting Author unless you are acting as an employee on behalf of your employer or a postgraduate student of an affiliated institution which is paying any applicable article publishing charge ("APC") for Open Access articles. Where the Submitting Author wishes to make the Work available on an Open Access basis (and intends to pay the relevant APC), the terms of reuse of such Open Access shall be governed by a Creative Commons licence – details of these licences and which Creative Commons licence will apply to this Work are set out in our licence referred to above.

Other than as permitted in any relevant BMJ Author's Self Archiving Policies, I confirm this Work has not been accepted for publication elsewhere, is not being considered for publication elsewhere and does not duplicate material already published. I confirm all authors consent to publication of this Work and authorise the granting of this licence.

For peer review only

Outcomes for university students following emergency care presentation for deliberate self-harm: a retrospective observational study of emergency departments in England for 2017/2018

Authors: Catherine Campbell, Research Assistant [a], Joe Dodd [a], Research Associate, Igor Francetic, Research Fellow [a]

Affiliation: [a] Health Organisation, Policy and Economics (HOPE) group, School of Health Sciences, University of Manchester, UK.

Correspondence to:
Igor Francetic
Health Organisation, Policy and Economics (HOPE) group, School of Health Sciences, University of Manchester, Williamson Building, Oxford Road, M13 9PL, Manchester, UK.
Email: igor.francetic@manchester.ac.uk.

Abstract

Objectives: Identify university-aged students and contrast their healthcare provision and outcomes with other patients in the same age group attending emergency departments for deliberate self-harm.

Design: Retrospective cross-sectional observational study.

Setting: Patients visiting 129 public hospital emergency departments across England between April 2017 and March 2018.

Participants: 14,074 patients aged 18-23 visiting emergency departments for conditions linked to deliberate self-harm, 1,016 of which were identified as university-aged students.

Outcome measures: We study various outcomes across the entire patient pathway in the emergency department: Waiting time to initial assessment upon arrival at the emergency department, count of investigations delivered, discharge destination (patients refusing treatment or leave before being seen, referred to another provider or admitted to inpatient care, discharged with no follow-up) and unplanned follow-up visit within 7 days.

Results: We find a statistically significant difference of 0.262 [-0.491,-0.0327] less investigations delivered to students compared to non-students (about 8 percent compared to the baseline number of investigations for non-students). Stratified analyses reveal that this difference is concentrated amongst students visiting the emergency department outside of regular working hours (-0.485 [-0.850,-0.120]) and students visiting for repeated deliberate self-harm episodes (-0.881 [-1.510,-0.252]). Unplanned reattendance within 7 days is lower among students visiting emergency departments during out of hours (-0.0306 [-0.0576,-0.00363]), whilst students arriving by ambulance are less likely to be referred to another provider (-0.0708 [-0.140,-0.00182]) compared to non-students.

Conclusions: We find evidence of less intense investigations being delivered to patients aged 18-23 identified as students compared to non-students visiting emergency departments after an episode of deliberate self-harm. Given the high risk of suicide attempts after episodes of deliberate self-harm among students, our findings may highlight the need for more focused interventions on this group of patients.

For peer review only

Word count: 4,445

Strengths and limitations of this study

- We propose an approximate approach to identify students in administrative records for emergency departments (EDs) in England, focusing on the population of attendances between April 2017 and March 2018.
- Supply-side confounders are accounted by a high-dimensional fixed effects, accounting for ED-specific combinations of day of the week and month of the year.
- We study outcomes across the entire pathway for patients attending EDs after episodes of deliberate self-harm, comparing students and non-students.
- The lack of a qualitative component in our study prevents a richer and more accurate interpretation of ED staffs' attitude towards self-harm patients.
- Variability in the systems and protocol to assess and treat self-harm presentations may limit comparability across geographic areas despite we account for hospital-specific fixed effects to circumvent these differences.

For peer review only

INTRODUCTION

Three-quarters of mental health disorders peak by the age of 25 years, an age-group associated with university attendance [1] and for which there is a large unmet need for mental health care [2]. As a result, there have been calls for increased research on student mental health [3]. Academic pressures and living far from social networks developed during early adolescence may pose additional stress on an already vulnerable group [4]. For instance, in the U.S, among 274 surveyed universities, 88 percent of counselling centre's directors reported an increase in severe psychological problems over the previous five years [5]. However, in England, trends in incidence of severe mental health problems and suicide do not seem to be higher among students compared to the general population of the same age [6–8]. Nevertheless, from a service provision perspective, the student population appears particularly vulnerable, especially in regards to limited availability and accessibility of mental health services for students [9–11].

Our study relates to literature surrounding the quality of care within emergency departments (EDs) for deliberate self-harm patients. Firstly, ED staff attitude can exert a crucial effect on the future health-seeking behaviours of self-harm patients [12]. This suggests that if a patient experiences a negative reaction to seeking help for their ailment, they are less likely to seek similar help again. Moreover, there is evidence patients who engage in self-harm or attempt suicide are found to be more negatively evaluated and viewed as "wasting resources" compared to patients attending for other reasons [13]. Timson et al. [14] further find a correlation between staff knowledge of mental health treatment and negativity towards self-harm patients. Finally, there is ample evidence relating hospital attendance for non-fatal self-harm to subsequent risk of completed and attempted suicide, indicating that treatment for self-harm attendances is particularly sensitive [15–18]. These studies highlight the potential impact of staff attitudes and perception of mental health on care outcomes for students attending ED for deliberate self-harm.

This paper builds on a growing body of literature regarding student mental health at university. Bolotnyy et al. [19] find that PhD economic students at top-ranked American universities have experienced depressive symptoms at a rate of over two times the population average, with only a small percentage receiving help. Levels of depressive symptoms are even higher among graduate students attending European universities [20]. These studies demonstrate the prevalence and severity of mental health in universities, common across countries. As mentioned, demand-side barriers such as misperceptions about care-seeking may contribute to the treatment gap among university students [21,22]. In light of this, Acampora et al. [23] find that amongst individuals who receive information about the benefits of seeking help, females present a higher likelihood of seeking help, resulting in improved mental health scores at follow-up. Holt and Powell [24] further find that 80 percent of university student attendees are not registered with a GP. This may reflect in an apparent misuse of emergency care services, which has been attributed to students failing to seek healthcare on campus due to fear of vulnerability and breached confidentiality [25].

Building on these stylised facts, we aim to explicitly study the healthcare provision and outcomes of university-aged students attending EDs for deliberate self-harm (DSH), contrasting them with other DSH patients. In doing so, given that administrative hospital records do not include information about student status, we propose a proxy indicator to identify students in Hospital Episode Statistics (HES) records.

For peer review only

METHODS

Data and sample

Our study builds on the attendance records of patients visiting EDs across England between April 2017 and March 2018. These records were obtained from the Hospital Episode Statistics (HES) [26]. HES contains routinely collected data covering procedures and treatments for all patients visiting secondary care facilities across England, tracking the patients' pathway from attendance to discharge. Attendance records contain an extensive range of information about patients, including the hospital site, admission and discharge dates and times, GP practice to which the patient is registered, and a comprehensive set of demographic information. HES also captures the patient's small geographical area of residence. This is at the Lower layer Super Output Area (LSOAs) level, which includes approximately 1,500 individuals. These are used to link information on area-level of deprivation, namely the income component of the Index of Multiple Deprivation [27].

Our target population is represented by patients aged 18 to 23 visiting EDs in England between April 2017 and March 2018, whose attendance was related to an episode of DSH (n=22,506). We focus on this period because of limitations in access to more recent data. Incidentally, this protects our analysis from any disruptions to services caused by the COVID-19 pandemic.

To obtain our analytical sample, we exclude attendances without a recorded LSOA (n=748). We also exclude records with missing values on key outcome or control variables (n=6,349), and with implausible waiting time values (zero total attendance time or with waiting times recorded at the maximum value allowed by the system, n=507). To avoid ambiguity, we also exclude the few patients appearing more than once on the same day in the dataset (n=115). Furthermore, we exclude patients attending Type 2 or Type 3 EDs (n=713), hence focusing only on visits to Type 1 EDs; that is 24-hour consultant-led services with full facilities for resuscitating patients. We do this as Type 2 and Type 3 EDs tend to have a different and less severe mix of patients [28]. Our final analytical sample is composed by 14,074 attendances categorised as related to episodes of deliberate self-harm, across 127 EDs in England, between April 2017 and March 2018. In Table 1 below we show descriptive statistics of our main covariates for the entire sample and for the two subgroups defined by our exposure.

For peer review only - http://bmjopen.bmj.com/site/about/guidelines.xhtml

From the Office for National Statistics, we obtain data on the demographic structure of LSOAs. We use this to identify areas with a disproportionately higher share of individuals aged 18-25, an age-range consistent with being a university student. Appendix 1 shows that the geographic distribution of these areas is aligned with the location of universities in England. From regular NHS Digital public releases [29] we obtained the number of patients registered with GP practices divided in five-year age groups. For each GP practice we computed the share of young patients aged between 14 and 44.

Exposure: Proxy indicator for university students visiting EDs in England

Our main exposure is represented by a proxy indicator which aims to identify university students visiting an ED in England based on HES Accident and Emergency (A&E) records. This proxy attempts to circumvent the absence of a specific variable indicating whether a patient is a student. Firstly, we define 18 to 23 as our target age range consistent with most university students. Secondly, we identify LSOAs where more than 10 percent of residents are aged between 18 and 25. This second criterion tries to identify areas in England where student residences are located (see Appendix 1). Thirdly, we identify GP practices whose patients are predominantly within an age range consistent with university students [30]. Specifically, we flagged 1,016 DSH attendance from patients aged 18 to 23 living in LSOAs likely to host students and registered with GP practices with over 50 percent of patients aged 14 to 44 (hence likely to treat students). This is equivalent to 7.22 percent of our sample.

Outcomes

Our analysis aims to study the healthcare provision and outcomes of university-aged students attending EDs for deliberate self-harm, contrasting them with other DSH patients in the same age range. The outcomes studied cover the entire patient pathway in ED from arrival to discharge. However, we do not focus on the choice of specific treatments delivered and their intensity as these are likely to depend on patient severity, which we are unable to measure. We firstly look at the time to initial assessment upon arrival at the ED. Secondly, we look at the count of investigations delivered. Thirdly, we look at various discharge destinations: patients refusing treatment or leave before being seen, referred to another provider or admitted to inpatient care, discharged with no follow-up. Finally, we measure unplanned follow-up visits within 7 days.

For peer review only - http://bmjopen.bmj.com/site/about/guidelines.xhtml

Empirical approach

A simple comparison in means across groups of patients by student status is unlikely to give an accurate account of differences in outcomes due to various sources of confounding. For example, patients flagged as students may have more or less severe conditions than other DSH patients. They may also systematically visit EDs that are intrinsically different or at times where EDs are more or less busy or staffed. To study whether being a student is associated with differences in outcomes and treatments compared to other DHS patients visiting an English ED, we conduct a regression-based analysis. Our main empirical specification reads as follows:

$$y_{ihmd} = \beta \times Student_i + \gamma \times Ind_i + \omega \times Att_{imd} + \delta\, Severity_{ihmd} + HDFE_{hmd} + \epsilon_{ihmd} \quad (1)$$

In equation (1), y_{ihmd} is the outcome observed for patient i visiting ED h on day d of month m. The vector Ind_i includes individual patient characteristics (age, ethnicity, whether lives in a rural area, income deprivation quintile of the LSOA of residence). The vector Att_{imd} includes characteristics of the patient in relation to the ED attendance, namely: mode of arrival, referral source, whether it is a repeated DSH episode, whether the attendance happens in-hours (between 6AM and 6PM) or out-of-hours (i.e. between 6PM and 6AM, during weekends or bank holidays [31]), and the volume of ED attendances on the same day. The volume of ED attendances on the same day accounts for differences in the level of pressure faced by ED staff, which may influence their behaviour towards patients. The primary ED diagnosis is used as a proxy for severity of DSH episode ($Severity_{ihmd}$). This concurs with ambulance arrivals to account for overall patient severity, which may impact how the DSH patient is treated in the ED. The list and frequencies of ED diagnoses is reported in Appendix 2. The vector HDFE represents a high-dimensional fixed effect (HDFE) that includes ED-specific combinations of month of year and day of week, in relation to the day of the ED attendance. The inclusion of a HDFE allows us to account for various supply and demand side observable and unobservable differences across EDs, and days of attendance, that may affect how DSH patients visiting an ED are treated [31]. In absence of more detailed information of staffing at the time of the attendance, this allows to account for shifts in EDs, that are typically arranged well in advance to accommodate known weekly and seasonal fluctuations in ED demand. Similarly, different EDs may have varied organisational structures and staff-skill mix; that is whether the staff are more or less at ease with patients visiting the department for an episode or DSH. Additionally, on the demand side, university students may be more likely to visits EDs for episodes of DSH at a specific time of year (e.g. during end of term exam periods) or location (e.g. hospitals located in major University cities).

Our main coefficient of interest is β associated with the exposure variable $Student_i$. This reflects the difference in outcomes attributable to being a student according to our proxy definition. In order to interpret β as an unbiased measure of our association of interest, we follow a standard selection on observables argument [32], assuming conditional mean independence. In other words, conditional on the set of characteristics and the high-dimensional fixed effects we control for, the error term ϵ_{ihmd} is mean independent of our student dummy.

We estimate our models with two related approaches. Firstly, for all outcomes we report estimates of naïve models. These explain outcomes only with our exposure using a Probit model for binary outcomes and a Poisson model for right-skewed outcomes. We then compare our naïve estimates to results from our preferred empirical specification, with and without the more detailed controls for patient severity, represented by primary ED diagnosis. For binary models we assume a linear probability model and estimate equation (1) using a feasible and efficient linear estimator, which accommodates high-dimensional fixed effects implemented in the Stata command *reghdfe* [33]. To account for the skewed distribution of waiting times and the number of investigations and procedures, we estimate models for these outcomes using a Poisson regression with a similar feasible efficient estimator designed to accommodate high-dimensional fixed effects implemented in the Stata command *ppmlhdfe* [34]. For Poisson models we report results as average marginal effects [35].

Sensitivity analyses

To further explore the relationship between our student proxy and outcomes in ED for DSH attendances we propose two stratified analyses. Firstly, we estimate separately models for attendances during (a) standard working hours and (b) out-of-hours respectively, as these periods may differ in the level of ED preparedness and resources. Secondly, we estimate two sets of models for (c) patients brought in by ambulance and (d) arrived at the ED by other means. The rationale for this is that patients arriving by ambulance are likely to have a higher priority. Finally, we estimate the models separately for patients visiting for a first or a repeated DSH episode during our study period. Unfortunately, we are not able to identify repeated DSH episodes before April 2017 due to data limitations. We estimate these sensitivity analyses using our preferred specification including control variables. We are unable to also control for primary ED diagnosis due to the emergence of singleton observations when partialling out the high-dimensional fixed when limiting sample size by splitting observations in sub-groups.

Patient and public involvement

This research did not involve members of the public or patients at any stage, beginning with developing the research questions to identifying the study design or outcomes. We did not consult members of the public in the interpretation of results, nor writing or editing the paper. We also have no specific plans to involve the public in the dissemination of the research findings. Nevertheless, Catherine Campbell developed this research whilst being enrolled as undergraduate student, i.e. as a member of the main target group of this study.

For peer review only - http://bmjopen.bmj.com/site/about/guidelines.xhtml

RESULTS

Seasonality of deliberate self-harm ED attendances

As mentioned in the methods section, there may be seasonality in the attendance of EDs for deliberate self-harm patients. Figure 1 below shows the seasonal variability in the share of patients attending EDs for an episode of deliberate self-harm (averaged across the 127 EDs considered in our analysis), for patients identified as students and non-students.

The data suggest a that approximately 1.1 to 1.4 percent of all monthly ED attendances are related to DSH episodes. For patients identified as students there is a clearer seasonality, with higher peaks in October and January, and lower prevalence compared to non-students during the summer months.

Descriptive statistics

Table 1 below shows characteristics of individuals in the sample, based on our main exposure. Patients aged 18-23 identified as students were more likely to be female (73.03 percent compared to 63.00 percent for non-students) and less likely to have a White ethnic background (86.52 percent compared to 93.87 percent for non-students). Students were also less likely to live in rural and deprived areas. There are no substantial differences between students and non-students across the other covariates.

In Table 2 we report summary statistics of outcomes by exposure status, with differences and corresponding test for difference in means and proportions. On average, patients aged 18-23 identified as students wait about 3.03 minutes longer for their initial assessment and receive 0.3 less investigations compared to non-students. They also seem to be less likely to be referred to another provider, to be admitted to hospital, and to have a follow-up visit scheduled at discharge. However, these descriptive differences may be confounded by various observed and unobserved differences between these two groups of young DSH patients.

For peer review only - http://bmjopen.bmj.com/site/about/guidelines.xhtml

Table 1. Descriptive statistics for attendances for the full sample and by exposure status

	Full sample		Not flagged as students		Flagged as students	
Variable	Perc.	Count	Perc.	Count	Perc.	Count
Female	64.65	9,099	64.00	8,357	73.03	742
Male	35.35	4,975	36.00	4,701	26.97	274
Ethnicity						
Any White background	93.34	13,136	93.87	12,257	86.52	879
Any Black background	1.45	204	1.33	174	2.95	30
Any Indian background	2.24	315	2.04	266	4.82	49
Any mixed background	2.98	419	2.76	361	5.71	58
Living in rural area	3.20	450	3.36	439	1.08	11
IMD (income component) deprivation quintile						
Least deprived	12.47	1,755	11.26	1,470	28.05	285
2nd quintile	14.22	2,002	14.28	1,865	13.48	137
3rd quintile	19.19	2,701	19.02	2,484	21.36	216
4th quintile	23.58	3,319	23.66	3,089	22.64	230
Most deprived	30.53	4,297	31.78	4,150	14.47	147
ED arrival mode						
Ambulance	48.98	6,893	49.14	6,417	46.85	476
Other	50.67	7,131	50.48	6,592	53.05	539
Not known	0.36	50	0.38	49	0.10	1
Referral source						
Self-referred	51.09	7,191	51.01	6,661	52.17	530
GP	1.58	222	1.53	200	2,17	22
Emergency services	31.04	4,368	31.38	4,097	26.67	271
Health care providers	8.50	1,196	8.29	1,083	11.12	113
Work or education	0.48	68	0.47	62	0.59	6
Other	7.31	1,029	7.31	955	7.28	74
Repeated DSH episodes	35.00	4,926	35.35	4,616	30.51	310
Out of hours (6AM-6PM) attendance	58.18	8,188	57.92	5,495	61.52	625
	Mean (SD)	Range	Mean (SD)	Range	Mean (SD)	Range
Daily ED attendances	416.88 (184.94)	88-1497	412.44 (184.16)	88-1497	474.00 (185.61)	145-1350
Age	20.28 (1.71)	18-23	20.26 (1.72)	18-23	20.43 (1.51)	18-23
Observations	14,074		13,058		1,016	

Notes: (1) The table shows percentages and counts for categorical or binary outcomes and mean/SD/range for discrete variables at the bottom. (2) The summary statistics refer to the full sample of DSH patients aged 18-23 (first column), and to the sub-samples identified as non-students (second column) and students (third column) based on our student proxy described in the "Methods" section.

Table 2. Summary of outcomes by exposure status

	Not flagged as students				Flagged as students				Difference	
Discrete outcomes	N	Mean	SD	Range	N	Mean	SD	Range	Diff.	*t*
Time to initial assessment	13,058	18.94	21.74	0-80	1,016	21.97	22.74	0-80	3.03***	4.42
Number of investigations	13,058	3.19	3.38	0-12	1,016	2.89	3.40	0-12	-0.29**	-2.67
Binary outcomes	N	Perc.	Count		N	Perc.	Count		Diff.	*z*
Refused or left before treatment	13,058	6.03	787		1,016	5.81	59		-0.22	-0.28
Referred to another provider	12,509	42.06	5,137		978	34.25	335		-6.81***	-4.17
Admitted to hospital	13,058	31.92	4,168		1,016	26.28	267		-5.64***	-3.73
Discharged with no follow-up	12,914	35.27	4,555		1,010	41.09	415		5.82***	3.72
Unplanned follow-up within 7 days	13,058	6.46	844		1,016	4.92	50		-1.54	-1.94

For peer review only

Notes: (1) The table shows percentages and counts for categorical or binary outcomes, and mean/SD/range for discrete outcomes at the top. (2) third column includes the difference between students and non-students, alongside the corresponding t statistic testing the difference in means or z statistic testing the difference in proportions. section. Stars indicate statistical significance as follows: * $p < 0.05$, ** $p < 0.01$, *** $p < 0.001$

For peer review only

Main results

Table 3 presents the estimated difference attributable to being a student for several indicators of ED performance, compared to non-students. We find a sizeable difference in the number of investigations delivered of -0.262 [-0.491,-0.0327] during the visit (about 8 percent of the baseline average number of investigations for non-students). This difference is consistent across the three empirical specification used (naïve model in column 1, adding patient characteristics and daily volume of attendances at the ED in column 2, and further accounting for differences in severity by controlling for the primary ED diagnosis in column 3). Our multivariate regression analysis suggests that all other differences emerging in the descriptive comparison in Table 2 are likely confounded by other factors.

Table 3. Main results on full sample

		(1)	(2)	(3)
Time to initial assessment	*Estimate*	3.032	-0.958	-1.033
	95% CI	[-1.207,7.271]	[-2.050,0.133]	[-2.130,0.0631]
	N	14074	11581	11579
Number of investigations	*Estimate*	-0.295	-0.402**	-0.262*
	95% CI	[-0.706,0.116]	[-0.680,-0.125]	[-0.491,-0.0327]
	N	14074	11294	11289
Refused or left before treatment	*Estimate*	-0.00220	-0.0101	-0.00914
	95% CI	[-0.0211,0.0167]	[-0.0308,0.0106]	[-0.0262,0.00790]
	N	14074	11852	11852
Referred to another provider	*Estimate*	-0.0681	-0.0273	-0.0107
	95% CI	[-0.143,0.00699]	[-0.0621,0.00753]	[-0.0529,0.0315]
	N	13487	11261	11261
Admitted to hospital	*Estimate*	-0.0564	-0.0235	-0.00143
	95% CI	[-0.118,0.00544]	[-0.0610,0.0140]	[-0.0437,0.0408]
	N	14074	11852	11852
Discharged with no follow-up	*Estimate*	0.0582	0.00601	-0.000976
	95% CI	[-0.0522,0.169]	[-0.0307,0.0427]	[-0.0408,0.0388]
	N	13924	11715	11715
Unplanned follow-up within 7 days	*Estimate*	-0.0154	-0.0204	-0.0221
	95% CI	[-0.0367,0.00584]	[-0.0440,0.00310]	[-0.0456,0.00145]
	N	14074	11852	11852
Controls		No	Patient characteristics<br>Daily att. volume at ED	Patient characteristics<br>Daily att. volume at ED<br>Primary ED diagnosis

Notes: (a) All models and 3 include the following covariates: gender (female or male), age, ethnicity, whether lives in a rural area, income deprivation quintile of the LSOA of residence, mode of arrival, referral source, whether it is a repeated DSH episode, whether the attendance happens in-hours (between 6AM and 6PM) or out-of-hours (between 6PM and 6AM), the volume of ED attendances on the same day at the same ED, primary ED diagnosis. (b) Models were estimated with a Poisson feasible efficient estimator designed to accommodate high-dimensional fixed effects (Correia et al., 2020) for time to initial assessment and number of investigations. For all other outcomes we estimated a linear probability model using a similar feasible efficient linear estimator accommodating high-dimensional fixed effects (Correia, 2016); (c) Estimates represent average marginal effects for the difference between being flagged as students and non-student, 95% confidence intervals in parentheses for standard errors clustered at the ED level. Stars indicate statistical significance as follows: * $p < 0.05$, ** $p < 0.01$, *** $p < 0.001$.

For peer review only - http://bmjopen.bmj.com/site/about/guidelines.xhtml

Table 4. Stratified analyses

		In-hours (1)	Out-of-hours (2)	Arrived by ambulance (3)	Did not arrive by ambulance (4)	First DSH episode (5)	Repeated DSH episode (6)
Time to initial assessment	*Estimate*	-1.511	-0.820	-1.997*	1.611	-1.007	-1.653
	95% CI	[-3.875,0.852]	[-2.339,0.699]	[-3.703,-0.292]	[-1.684,4.905]	[-2.754,0.739]	[-4.206,0.900]
	N	3648	5758	4436	4734	6556	3013
Number of investigations	*Estimate*	-0.387	-0.485**	-0.545***	-0.474*	-0.259	-0.881**
	95% CI	[-0.804,0.0295]	[-0.850,-0.120]	[-0.839,-0.250]	[-0.844,-0.104]	[-0.577,0.0601]	[-1.510,-0.252]
	N	3581	5548	4533	4536	6386	2876
Refused or left before treatment	*Estimate*	-0.00461	-0.0157	-0.0166	-0.00294	-0.0111	-0.00441
	95% CI	[-0.0341,0.0248]	[-0.0433,0.0119]	[-0.0468,0.0135]	[-0.0298,0.0239]	[-0.0412,0.0191]	[-0.0360,0.0272]
	N	3779	5910	4728	4973	6733	3125
Referred to another provider	*Estimate*	-0.0334	-0.0140	-0.0708*	-0.0230	-0.0202	-0.0539
	95% CI	[-0.109,0.0425]	[-0.0585,0.0306]	[-0.140,-0.00182]	[-0.0837,0.0377]	[-0.0633,0.0229]	[-0.169,0.0614]
	N	3553	5603	4459	4707	6402	2922
Admitted to hospital	*Estimate*	-0.00424	-0.00631	-0.0602	-0.0119	-0.0195	0.00206
	95% CI	[-0.0762,0.0678]	[-0.0476,0.0350]	[-0.127,0.00680]	[-0.0760,0.0522]	[-0.0534,0.0144]	[-0.0891,0.0932]
	N	3779	5910	4728	4973	6733	3125
Discharged with no follow-up	*Estimate*	-0.00424	0.00450	0.0276	-0.0122	0.0260	-0.0336
	95% CI	[-0.0762,0.0678]	[-0.0442,0.0532]	[-0.0258,0.0811]	[-0.0641,0.0397]	[-0.0156,0.0675]	[-0.117,0.0494]
	N	3779	5829	4681	4885	6661	3070
Unplanned follow-up within 7 days	*Estimate*	-0.00782	-0.0306*	-0.0182	-0.0211	-0.0179	-0.0442
	95% CI	[-0.0605,0.0449]	[-0.0576,-0.00363]	[-0.0463,0.00997]	[-0.0533,0.0110]	[-0.0556,0.0199]	[-0.146,0.0573]
	N	3728	5910	4728	4973	6733	3125

Notes: (a) All models and 3 include the following covariates: gender (female or male), age, ethnicity, whether lives in a rural area, income deprivation quintile of the LSOA of residence, mode of arrival, referral source, whether it is a repeated DSH episode, whether the attendance happens in-hours (between 6AM and 6PM) or out-of-hours (between 6PM and 6AM), the volume of ED attendances on the same day at the same ED. (b) Models were estimated with a Poisson feasible efficient estimator designed to accommodate high-dimensional fixed effects (Correia et al., 2020) for time to initial assessment and number of investigations. For all other outcomes we estimated a linear probability model using a similar feasible efficient linear estimator accommodating high-dimensional fixed effects (Correia, 2016); (c) Estimates represent average marginal effects for the difference between being flagged as students and non-student, 95% confidence intervals in parentheses for standard errors clustered at the ED level. Stars indicate statistical significance as follows: * $p < 0.05$, ** $p < 0.01$, *** $p < 0.001$.

For peer review only - http://bmjopen.bmj.com/site/about/guidelines.xhtml

Sensitivity analysis

In Table 4 we report estimates for our specification including control variables (but not primary ED diagnosis) for our three sensitivity checks, namely the stratified analyses by arrival time (in-hours vs. out-of-hours), arrival mode (patients brought in by ambulance vs. arriving at the ED by other means) and first or repeated DSH episode. Our main finding of fewer investigations delivered to patients identified as students seems concentrated amongst out-of-hours visits (column 2) and patients visiting for repeated DSH episodes (column 6, as opposed to the first DSH episode in the study period in column 5). Patient visiting during nights, weekends or bank holidays are also less likely to have an unplanned follow-up visit within 7 days (-0.0306 [-0.0576,-0.00363]).

Columns 3 and 4 reflect the differences in outcomes attributable to whether the patient arrived by ambulance, comparing students and non-students. Whilst the main results in Table 3 account for the mode of arrival, splitting DSH patients by ambulance arrivals may help to better account for an intrinsic unobserved heterogeneity between these patient groups. We find that students who arrive by ambulance wait about two less minutes for initial assessment (-1.997 [-3.703,-0.292]) and are about 7 percent less likely (-0.0708 [-0.140,-0.00182]) to be referred to another provider. The number of investigations performed at a patient's initial assessment for both arrival modes is consistent with our main results.

DISCUSSION

Summary of findings

Our analysis proposes a method to identify patients who are likely students in the HES ED records, and explores differences in healthcare outcomes between students and non-students attending an ED after an episode of deliberate self-harm. We find a statistically significant difference of 0.262 less investigations delivered to students compared to non-students, which is equivalent to about 8 percent compared to the baseline number of investigations for non-students. All other differences in ED care outcomes are not statistically significant in our main analysis. The results of our stratified analyses by time of attendance (regular times compared to out of hours, i.e. 6PM to 6AM and weekends), ambulance arrival, and first vs. repeated DSH episode confirm the main findings providing a more nuanced picture. The reduced number of investigations (compared to non-students) is concentrated amongst students visiting EDs during out-of-

For peer review only

hours and for repeated DSH episodes. Students visiting during out-of-hours are also less likely to have unplanned follow-up visits within 7 days. On the other hand, students arriving at the ED by ambulance seem to wait slightly less for initial assessment and be less likely to be referred to another provider.

Strengths and weaknesses of the study

This paper has several strengths. Firstly, our analysis uses a large dataset capturing ED attendances of DSH patients in English hospitals for an entire year. Secondly, it trials a student proxy indicator to identify and measure differences in emergency care outcomes compared to other patients attending EDs for the same reason – deliberate self-harm. Thirdly, in order to isolate the association between ED care outcomes and being a student, we address potential sources of confounding both on the demand (i.e. patients) and supply (i.e. hospital) sides. To account for compositional differences and unobserved patient severity, our main specification controls for patient characteristics and primary ED diagnosis. On the supply side, existing literature finds that – based on historical attendance patterns – some variation in attendance volume is predictable and as a result EDs are capable of adjusting capacity accordingly [36]. To circumvent the confounding effect on estimates resulting from ED shift scheduling patterns and differing times of attendance, we control for number of ED attendances on the same day and include an ED-specific high-dimensional fixed effect to clean our estimates from expected patterns of shift planning and natural variations in ED attendance across areas and seasons.

Despite our range of sensitivity checks, some limitations remain. First, our student indicator is only a proxy, and therefore, be interpreted with caution. The marked difference in seasonality of DSH episodes (Figure 1) and the geographical location of students' areas of residence (Appendix 1) provide some validation. Nevertheless, our method of identifying students is conservative, suggesting our results are likely interpretable as lower bounds of student attendance volumes and related differences in ED care. Moreover, our study does not include any qualitative component; we do not interview ED staff members in regards to their attitude towards self-harm patients, a practice which would help to interpret some of our findings [12]. Finally, variability in the systems and protocol to assess and treat self-harm presentations may impede a meaningful comparison across areas [37]. We try to circumvent this latter aspect by accounting for hospital-specific fixed effects, and by exploring the heterogeneity in the results across areas more or less exposed to, and equipped for, episodes of deliberate self-harm.

For peer review only - http://bmjopen.bmj.com/site/about/guidelines.xhtml

Strengths and weaknesses in relation to other studies

A related study by Chartrand et al. [38] proposes a longitudinal analysis of differences in discharge destination and rate of reattendance between patients who experience DSH and those who attempt suicide. They find those who re-attended for DSH and suicidal attempt were more likely to be discharged to usual care during the initial assessment compared to a patient only attending for DSH. In view of this, a potential weakness of our study – dictated by data limitations – is that we did not include an indicator for suicide attempts. This is an important factor as patients who engage in DSH are at greater risk of subsequently dying by suicide [16]. Our results were consistent with a paper by Sinclair et al. [39] whom employs a crossover methodological design to examine the differences in care before and after the introduction of a dedicated psychiatric nurse service. Importantly, the author finds an increase in the accuracy of assessment and diagnosis post-intervention, wherein nurses are better trained to address DSH patients during initial assessments. These results may be consistent with a higher effectiveness in assessing patients, and hence explain the presented negative association between patient characteristics (being a student attending for DSH) and the number of investigations.

University students are not a homogenous group; rather there is large variation in health-seeking behaviours within the student population and compared to other patients in the same age group. Existing literature find that upon arrival at the ED, students experience feelings of shame and fear surrounding a perceived 'failure to cope' and lack of confidentiality [40,41]. It is plausible that these feelings are exacerbated when attending outside of normal working hours, pushing these patients to form a preference for not reattending the ED in case of need [42].

The decreased number of investigations amongst students, compared to non-students, is consistent with physician's actions being guided by a both a priority to treat physical symptoms (over psychological symptoms) and limited knowledge in terms of the immediate management of self-harm patients. In particular, qualitative literature finds self-harm patients experience negative attitudes from staff and differences in treatment compared to other patients [43]. These patterns may originate from an increased level of uncertainty in dealing with this patient group, wherein a deficit in self-harm training has be emphasised [44]. Previous literature has also raised concerns over a lack of local policies that prioritise mental health over physical health [45]. Finally, the larger difference in the number of investigations for students visiting EDs for repeated deliberate self-harm episodes may reflect either a better specific

For peer review only

knowledge of the specific patient by ED staff or a marker of a generalised attitude toward students frequently attending EDs for mental health related problems materialising in episodes of self-harm [12,13].

In addition, the reduced number of investigations recorded amongst students compared to non-students irrespective of the arrival mode (i.e. ambulance or not) may reflect faults in the ED triage process. The first step upon arrival requires patients to provide a brief summary of their health condition(s) at reception. Following this, patients wait for an initial assessment, wherein a medical or nursing professional determines priority for treatment by taking a brief medical history, a pain assessment and any collection of early warning scores [46]. Therefore, if staff are not trained in the management of mental health patients, EDs overlook a crucial opportunity to diagnose mental illness and commence a treatment pathway that may prevent subsequent self-harming behaviour [47].

Policy implications

Poor mental health is a major clinical priority given that it is at least partially preventable and a contributing risk factor for suicide attempts, especially among teens and young adults [15]. Our results have implications for both university health care and ED systems. English universities need to further invest in resources to address the heightened demands for counselling services [48]. Oversubscription and a shortage of trained staff have impacted services through reduced length and number of sessions, longer waitlists, and more referrals to off-campus providers [2]. In addition, university health centres need to prioritise information campaigns to decrease the stigma surrounding self-harm that prevents many students from seeking help, and consequently, lead to EDs being a first point of contact. The higher probability of refusing or leaving without treatment when attending EDs in-hours suggests the need for greater access to dedicated mental health staff or treatment, better access to community-based resources, increased availability of inpatient psychiatric beds and a separate safe space in the ED [49]. In addition, roughly one half of DSH patients in ED do not receive a psychosocial assessment [50], which further indicates the need to regulate and standardise mental health services. This suggest that better coordination between university mental health services and EDs may be needed in the long-term. In the short-term, in view of existing time and resource constraints, educating existing staff in the evaluation and management of DSH patients may be the only feasible approach to address such issues.

Open questions and future research

For peer review only

This paper could be advanced in several ways. Further research is needed to investigate the association between students and ED outcomes amongst those who have current contact with mental health services at the time of self-harm. Future studies should aim to understand differences in student's health-seeking behaviour that could help design policies to mitigate staff bias towards this sub-group. Future research should also focus on the scarcity of integrated inpatient and outpatient mental health services at the county level. This is crucial in providing unified health care for a high-risk and vulnerable population, whilst also reducing capacity constraints on EDs.

For peer review only

Contributors: CC and IF developed the original idea for the study, designed the analysis and carried out the analysis. CC, IF and JD contributed substantially to the interpretation of results and the writing of the manuscript. IF is responsible for the overall content as the guarantor.

Funding: This research was supported by the National Institute for Health Research (NIHR) Applied Research Collaboration Greater Manchester (NIHR200174).

Competing interests: None to declare.

Data availability statement: The Hospital Episode Statistics data that support the findings of this study are available upon request from NHS Digital. Restrictions apply to the availability of these data. They were used under licence for this study only, and so are not publicly available.

Patient consent for publication: Not applicable.

Ethics approval: The study is based on routinely collected secondary data. The study did not require a specific ethical approval because no study participants were put at risk during the study and confidentiality was maintained by the use of pseudonymised data provided by the Hospital Episodes Statistics. No informed consent was required as no identifiable patient data were collected.

Acknowledgements: We are grateful for comments and suggestions received from colleagues during Health Organisation, Policy and Economics (HOPE) seminars at the University of Manchester. Additional thanks to the Winter 2022 Health Economics Study Group (HESG) conference participants in Leeds for feedback during the poster session.

For peer review only

References

1 Kessler RC, Amminger GP, Aguilar-Gaxiola S, *et al.* Age of onset of mental disorders: a review of recent literature. *Curr Opin Psychiatry*. 2007;20:359–64.
2 The WHO World Mental Health Survey Consortium. Prevalence, Severity, and Unmet Need for Treatment of Mental Disorders in the World Health Organization World Mental Health Surveys. *JAMA*. 2004;291:2581–90.
3 Dodd AL. Student mental health research: moving forwards with clear definitions. *J Ment Health*. 2021;30:273–5.
4 Storrie K, Ahern K, Tuckett A. A systematic review: Students with mental health problems—A growing problem. *Int J Nurs Pract*. 2010;16:1–6.
5 Gallagher RP. Thirty Years of the National Survey of Counseling Center Directors: A Personal Account. *J Coll Stud Psychother*. 2012;26:172–84.
6 Gunnell D, Caul S, Appleby L, *et al.* The incidence of suicide in University students in England and Wales 2000/2001–2016/2017: Record linkage study. *J Affect Disord*. 2020;261:113–20.
7 Macaskill A. The mental health of university students in the United Kingdom. *Br J Guid Couns*. 2013;41:426–41.
8 McManus S, Gunnell D. Trends in mental health, non-suicidal self-harm and suicide attempts in 16–24-year old students and non-students in England, 2000–2014. *Soc Psychiatry Psychiatr Epidemiol*. 2020;55:125–8.
9 Querstret D. Collaborating with Students to Support Student Mental Health and Well-being. In: Lygo-Baker S, Kinchin IM, Winstone NE, eds. *Engaging Student Voices in Higher Education : Diverse Perspectives and Expectations in Partnership*. Cham: Springer International Publishing 2019:191–207. https://doi.org/10.1007/978-3-030-20824-0_12
10 Wilson C, Spencer G. The mental health support needs of university students with long-term physical health conditions. *Health Educ*. 2022;122:584–98.
11 Yorgason JB, Roper SO, Sandberg JG, *et al.* Stress spillover of health symptoms from healthy spouses to patient spouses in older married couples managing both diabetes and osteoarthritis. *Fam Syst Health*. 2012;30:330–43.
12 Mackay N, Barrowclough C. Accident and emergency staff's perceptions of deliberate self-harm: Attributions, emotions and willingness to help. *Br J Clin Psychol*. 2005;44:255–67.
13 Cleaver K. Attitudes of emergency care staff towards young people who self-harm: A scoping review. *Int Emerg Nurs*. 2014;22:52–61.
14 Timson D, Priest H, Clark-Carter D. Adolescents who self-harm: Professional staff knowledge, attitudes and training needs. *J Adolesc*. 2012;35:1307–14.
15 Beckman K, Mittendorfer-Rutz E, Waern M, *et al.* Method of self-harm in adolescents and young adults and risk of subsequent suicide. *J Child Psychol Psychiatry*. 2018;59:948–56.
16 Bergen H, Hawton K, Waters K, *et al.* How do methods of non-fatal self-harm relate to eventual suicide? *J Affect Disord*. 2012;136:526–33.
17 Chan MKY, Bhatti H, Meader N, *et al.* Predicting suicide following self-harm: systematic review of risk factors and risk scales. *Br J Psychiatry*. 2016;209:277–83.
18 Hawton K, Bergen H, Kapur N, *et al.* Repetition of self-harm and suicide following self-harm in children and adolescents: findings from the Multicentre Study of Self-harm in England. *J Child Psychol Psychiatry*. 2012;53:1212–9.
19 Bolotnyy V, Basilico M, Barreira P. Graduate Student Mental Health: Lessons from American Economics Departments. *J Econ Lit*. 2022;60:1188–222.
20 Macchi E, Sievert C, Bolotnyy V, *et al.* Mental Health in European Economics Departments. 2023. https://doi.org/10.2139/ssrn.4509725
21 Eisenberg D, Golberstein E, Gollust SE. Help-Seeking and Access to Mental Health Care in a University Student Population. *Med Care*. 2007;45:594–601.
22 Ziano I, Koc Y. Prototypes of People With Depression. *Psychol Sci*. 2023;34:1295–308.
23 Acampora M, Capozza F, Moghani V. Mental Health Literacy, Beliefs and Demand for Mental Health Support. 2022. https://doi.org/10.2139/ssrn.4261487

For peer review only

24 Holt M, Powell S. Healthy Universities: a guiding framework for universities to examine the distinctive health needs of its own student population. *Perspect Public Health*. 2017;137:53–8.
25 Tran D-MT, Silvestri-Elmore A. Healthcare-seeking behaviours in college students and young adults: a review. *J Res Nurs*. 2021;26:320–38.
26 NHS England. Hospital Episode Statistics (HES). NHS Digit. 2023. https://digital.nhs.uk/data-and-information/data-tools-and-services/data-services/hospital-episode-statistics (accessed 10 August 2023)
27 Department for Communities and Local Government. English indices of deprivation 2015. 2015. https://www.gov.uk/government/statistics/english-indices-of-deprivation-2015
28 The King's Fund. What's going on with A&E waiting times? 2020. https://www.kingsfund.org.uk/projects/urgent-emergency-care/urgent-and-emergency-care-mythbusters (accessed 11 May 2022)
29 NHS Digital. Patients Registered at a GP Practice. NHS Digit. 2022. https://digital.nhs.uk/data-and-information/publications/statistical/patients-registered-at-a-gp-practice (accessed 19 August 2022)
30 Anselmi L, Everton A, Shaw R, *et al.* Estimating local need for mental healthcare to inform fair resource allocation in the NHS in England: cross-sectional analysis of national administrative data linked at person level. *Br J Psychiatry*. 2020;216:338–44.
31 Turner AJ, Anselmi L, Lau Y-S, *et al.* The effects of unexpected changes in demand on the performance of emergency departments. *Health Econ*. 2020;29:1744–63.
32 Altonji JG, Elder TE, Taber CR. Selection on Observed and Unobserved Variables: Assessing the Effectiveness of Catholic Schools. *J Polit Econ*. 2005;113:151–84.
33 Correia S. A Feasible Estimator for Linear Models with Multi-Way Fixed Effects. 2016. http://scorreia.com/research/hdfe.pd
34 Correia S, Guimarães P, Zylkin T. Fast Poisson estimation with high-dimensional fixed effects. *Stata J*. 2020;20:95–115.
35 Norton EC, Dowd BE, Maciejewski ML. Marginal Effects—Quantifying the Effect of Changes in Risk Factors in Logistic Regression Models. *JAMA*. 2019;321:1304–5.
36 Anselmi L, Meacock R, Kristensen SR, *et al.* Arrival by ambulance explains variation in mortality by time of admission: retrospective study of admissions to hospital following emergency department attendance in England. *BMJ Qual Saf*. 2017;26:613–21.
37 Joyce M, Chakraborty S, O'Sullivan G, *et al.* National Self-Harm Registry Ireland Annual Report 2020. Cork: National Suicide Research Foundation 2022.
38 Chartrand H, Tefft B, Sareen J, *et al.* A Longitudinal Study of Correlates, Discharge Disposition, and Rate of Re-presentation to Emergency Services of Adults who Engage in Non-suicidal Self-injury. *Arch Suicide Res*. 2022;26:1141–58.
39 Sinclair L, Hunter R, Hagen S, *et al.* How effective are mental health nurses in A&E departments? *Emerg Med J*. 2006;23:687–92.
40 Owens C, Hansford L, Sharkey S, *et al.* Needs and fears of young people presenting at accident and emergency department following an act of self-harm: secondary analysis of qualitative data. *Br J Psychiatry*. 2016;208:286–91.
41 Mulhearne P, Cotter P, O'Shea M, *et al.* Experiences of registered general nurses who care for patients presenting with self-harm to the emergency department in Ireland. *Int Emerg Nurs*. 2021;58:101047.
42 Cleaver K, Meerabeau L, Maras P. Attitudes towards young people who self-harm: age, an influencing factor. *J Adv Nurs*. 2014;70:2884–96.
43 Gibson R, Carson J, Houghton T. Stigma towards non-suicidal self-harm: evaluating a brief educational intervention. *Br J Nurs*. 2019;28:307–12.
44 Wolf LA, Perhats C, Delao AM. US emergency nurses' perceptions of challenges and facilitators in the management of behavioural health patients in the emergency department: A mixed-methods study. *Australas Emerg Nurs J*. 2015;18:138–48.
45 Department of Health. A&E clinical quality indicators: Implementation guidance and data definitions. 2010. https://webarchive.nationalarchives.gov.uk/ukgwa/20130105030902mp_/http://www.dh.gov.uk/en/

For peer review only

Publicationsandstatistics/Publications/PublicationsPolicyAndGuidance/DH_122868 (accessed 5 January 2024)
46 Butler C, Joiner R, Bradley R, *et al.* Self-harm prevalence and ideation in a community sample of cis, trans and other youth. *Int J Transgenderism*. 2019;20:447–58.
47 Furber G, Segal L, Leach M, *et al.* Preventing mental illness: closing the evidence-practice gap through workforce and services planning. *BMC Health Serv Res*. 2015;15:283.
48 Prince JP. University student counseling and mental health in the United States: Trends and challenges. *Ment Health Prev*. 2015;3:5–10.
49 Cullen SW, Diana A, Olfson M, *et al.* If You Could Change 1 Thing to Improve the Quality of Emergency Care for Deliberate Self-harm Patients, What Would It Be? A National Survey of Nursing Leadership. *J Emerg Nurs*. 2019;45:661–9.
50 Bennewith O, Peters TJ, Hawton K, *et al.* Factors associated with the non-assessment of self-harm patients attending an Accident and Emergency Department: Results of a national study. *J Affect Disord*. 2005;89:91–7.

For peer review only

Figure caption

Figure 1. Share of DSH patients of all ED attendances by month

Note: The figure shows the overall share of DSH attendances in each month across all 127 EDs in our sample, split between patients aged 18-23 identified as students and non-students using the proxy outlined in the "Methods" section.

For peer review only - http://bmjopen.bmj.com/site/about/guidelines.xhtml

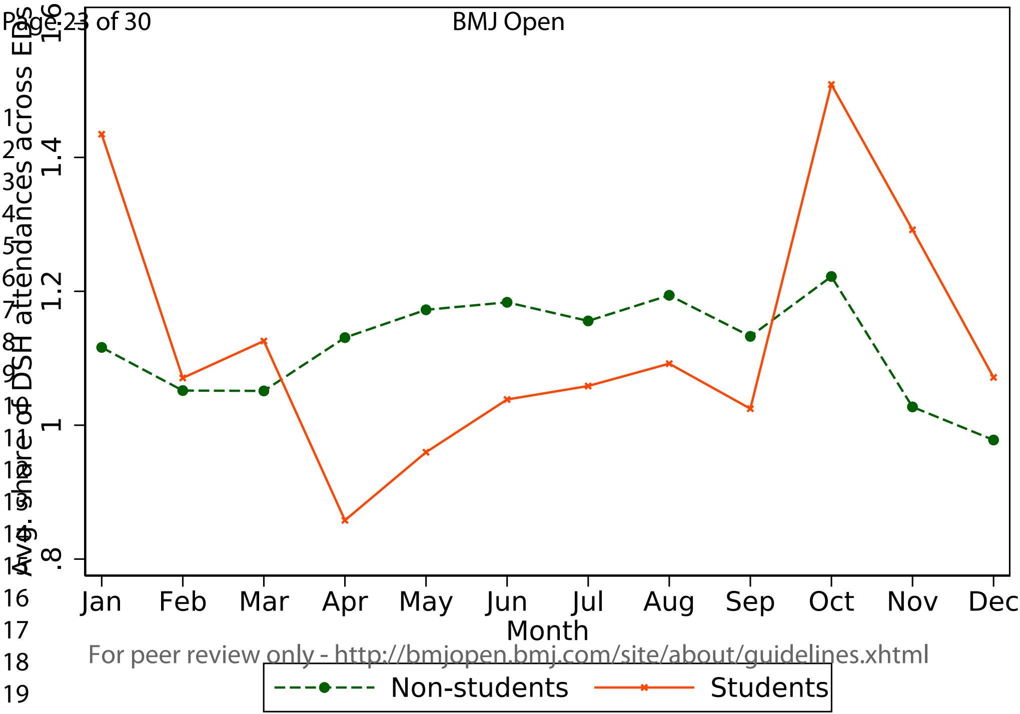
Avg. share of DSH attendances across EDs
1.6
1.4
1.2
1
.8
Jan
Feb
Mar
Apr
May
Jun
Jul
Aug
Sep
Oct
Nov
Dec
Month
Non-students
Students

Supplementary material to the paper titled “Outcomes for university students following emergency care presentation for deliberate self-harm: retrospective observational study of Emergency Departments in England for 2017/2018”

Authors: Catherine Campbell, Joe Dodd, Igor Francetic

Version: January 2024.

Appendix 1: Distribution of LSOAs with more than 10 percent of residents aged 18-23

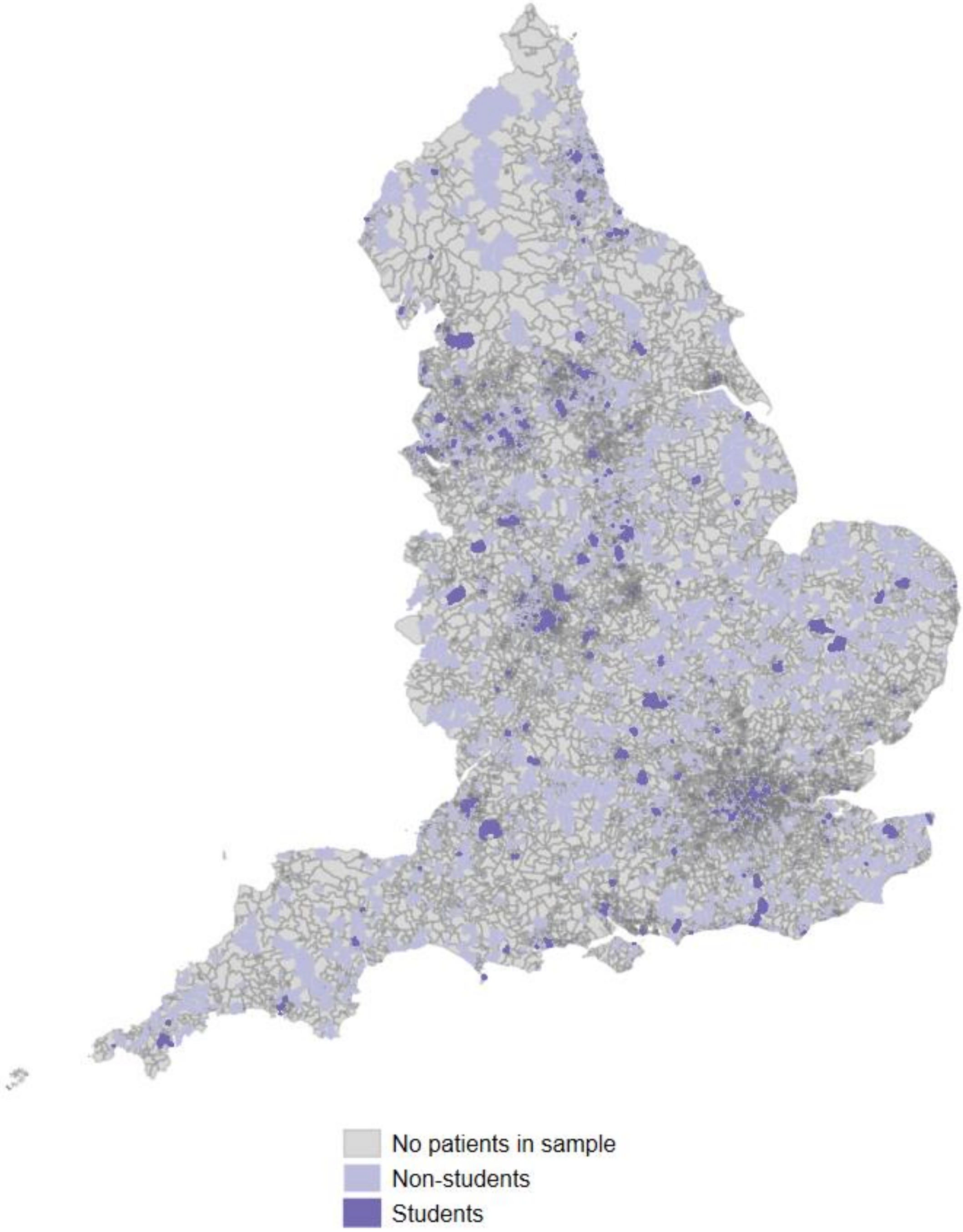

Note: Grey areas represent LSOAs in England that are not represented in our sample. Areas in light purple represent LSOAs of residence for patients not identified as students in our sample. Areas in darker purple represent LSOAs of residence of patients identified as students. The latter are clustered around university areas in England, for example Exeter, Bristol, Bath, Reading, London, Oxford, Cambridge, Birmingham, Coventry, Norwich, Liverpool, Manchester, Leeds, Sheffield, York, Lancaster, Durham and Newcastle among others.

Appendix 2: Distribution of primary ED codes (severity proxy) for analytical sample of attendances

Primary ED Diagnosis	Freq.	Percent
Laceration	1,244	8.84
Contusion/abrasion	360	2.56
Soft tissue inflammation	95	0.68
Head injury	108	0.77
Dislocation/fracture/joints	281	2.00
Sprain/ligament injury	107	0.76
Muscle/tendon injury	43	0.31
Burns and scalds	33	0.23
Electric shock	1	0.01
Foreign body	227	1.61
Bites/stings	13	0.09
Poisoning (inc overdose)	4,780	33.96
Near drowning	2	0.01
Visceral injury	6	0.04
Infectious disease	3	0.02
Local infection	24	0.17
Septicaemia	1	0.01
Cardiac conditions	28	0.20
Cerebro-vascular conditions	3	0.02
Other vascular conditions	2	0.01
Haematological conditions	4	0.03
Central nervous system conditions	43	0.31
Respiratory conditions	14	0.10
Gastrointestinal conditions	60	0.43
Urological conditions (inc cystitis)	12	0.09
Obstetric conditions	1	0.01
Gynaecological conditions	36	0.26
Diabetes and other endocrinological conditions	15	0.11
Dermatological conditions	6	0.04
Allergy (inc anaphylaxis)	3	0.02
Facio-maxillary conditions	2	0.01
ENT conditions	8	0.06
Psychiatric conditions	2,154	15.30
Ophthalmological conditions	17	0.12
Social problems (inc chronic alcoholism)	94	0.67
Diagnosis not classifiable	1,407	10.00
Nothing abnormal detected	189	1.34
Missclassified	703	5.00
No/missing diagnosis	1,945	13.82
Total	14,074	100

For peer review only

The RECORD statement – checklist of items, extended from the STROBE statement, that should be reported in observational studies using routinely collected health data.

	Item No.	STROBE items	Location in manuscript where items are reported	RECORD items	Location in manuscript where items are reported
**Title and abstract**					
	1	(a) Indicate the study's design with a commonly used term in the title or the abstract (b) Provide in the abstract an informative and balanced summary of what was done and what was found	Title and abstract – page 1.	RECORD 1.1: The type of data used should be specified in the title or abstract. When possible, the name of the databases used should be included.<br><br>RECORD 1.2: If applicable, the geographic region and timeframe within which the study took place should be reported in the title or abstract.<br><br>RECORD 1.3: If linkage between databases was conducted for the study, this should be clearly stated in the title or abstract.	Title and abstract – page 1.
**Introduction**					
Background rationale	2	Explain the scientific background and rationale for the investigation being reported	Introduction – page 1.		
Objectives	3	State specific objectives, including any prespecified hypotheses	Introduction, final paragraph – pages 1 & 2.		
**Methods**					
Study Design	4	Present key elements of study design early in the paper	Methods – pages 2-4.		
Setting	5	Describe the setting, locations, and relevant dates, including periods of recruitment, exposure, follow-up, and data collection	Methods, subsections: 'data and sample' (page 2) and 'exposure' (page 3).		

For peer review only

Participants	6	*(a) Cohort study* - Give the eligibility criteria, and the sources and methods of selection of participants. Describe methods of follow-up<br>*Case-control study* - Give the eligibility criteria, and the sources and methods of case ascertainment and control selection. Give the rationale for the choice of cases and controls<br>*Cross-sectional study* - Give the eligibility criteria, and the sources and methods of selection of participants<br><br>*(b) Cohort study* - For matched studies, give matching criteria and number of exposed and unexposed<br>*Case-control study* - For matched studies, give matching criteria and the number of controls per case	Methods, subsection 'data and sample' – page 2.	RECORD 6.1: The methods of study population selection (such as codes or algorithms used to identify subjects) should be listed in detail. If this is not possible, an explanation should be provided.<br><br>RECORD 6.2: Any validation studies of the codes or algorithms used to select the population should be referenced. If validation was conducted for this study and not published elsewhere, detailed methods and results should be provided.<br><br>RECORD 6.3: If the study involved linkage of databases, consider use of a flow diagram or other graphical display to demonstrate the data linkage process, including the number of individuals with linked data at each stage.	Methods, subsection 'data and sample' – page 2.
Variables	7	Clearly define all outcomes, exposures, predictors, potential confounders, and effect modifiers. Give diagnostic criteria, if applicable.	Methods, subsections 'exposure', 'outcomes' and 'empirical approach' – pages 3 & 4.	RECORD 7.1: A complete list of codes and algorithms used to classify exposures, outcomes, confounders, and effect modifiers should be provided. If these cannot be reported, an explanation should be provided.	Methods, subsection 'empirical approach' – pages 3 & 4.
Data sources/ measurement	8	For each variable of interest, give sources of data and details of methods of assessment (measurement).<br>Describe comparability of assessment methods if there is more than one group	Methods – pages 2-4.		

For peer review only

Bias	9	Describe any efforts to address potential sources of bias	Methods, subsection 'empirical approach' – page 4.		
Study size	10	Explain how the study size was arrived at	Methods, subsections 'data and sample' and 'exposure' – pages 2 & 3.		
Quantitative variables	11	Explain how quantitative variables were handled in the analyses. If applicable, describe which groupings were chosen, and why	Methods, subsection 'empirical approach' – pages 3 & 4.		
Statistical methods	12	(a) Describe all statistical methods, including those used to control for confounding<br>(b) Describe any methods used to examine subgroups and interactions<br>(c) Explain how missing data were addressed<br>(d) *Cohort study* - If applicable, explain how loss to follow-up was addressed<br>*Case-control study* - If applicable, explain how matching of cases and controls was addressed<br>*Cross-sectional study* - If applicable, describe analytical methods taking account of sampling strategy<br>(e) Describe any sensitivity analyses	Methods, subsections 'empirical approach' and 'sensitivity analyses' – pages 3 & 4.		
Data access and cleaning methods		..		RECORD 12.1: Authors should describe the extent to which the investigators had access to the database	Methods, subsection 'data and sample' – page 2.

For peer review only - http://bmjopen.bmj.com/site/about/guidelines.xhtml

				population used to create the study population.<br><br>RECORD 12.2: Authors should provide information on the data cleaning methods used in the study.	
Linkage		..		RECORD 12.3: State whether the study included person-level, institutional-level, or other data linkage across two or more databases. The methods of linkage and methods of linkage quality evaluation should be provided.	Methods, subsection 'data and sample' – page 2.
**Results**					
Participants	13	(a) Report the numbers of individuals at each stage of the study (*e.g.*, numbers potentially eligible, examined for eligibility, confirmed eligible, included in the study, completing follow-up, and analysed)<br>(b) Give reasons for non-participation at each stage.<br>(c) Consider use of a flow diagram	Methods, subsections 'data and sample' and 'exposure' – pages 2 & 3.	RECORD 13.1: Describe in detail the selection of the persons included in the study (*i.e.,* study population selection) including filtering based on data quality, data availability and linkage. The selection of included persons can be described in the text and/or by means of the study flow diagram.	Methods, subsections 'data and sample' and 'exposure' – pages 2 & 3.
Descriptive data	14	(a) Give characteristics of study participants (*e.g.*, demographic, clinical, social) and information on exposures and potential confounders<br>(b) Indicate the number of participants with missing data for each variable of interest<br>(c) *Cohort study* - summarise follow-up time (*e.g.*, average and total amount)	Results, subsection 'descriptive statistics' – pages 5 & 6.		

For peer review only

Outcome data	15	*Cohort study* - Report numbers of outcome events or summary measures over time<br>*Case-control study* - Report numbers in each exposure category, or summary measures of exposure<br>*Cross-sectional study* - Report numbers of outcome events or summary measures	Results, subsection 'descriptive statistics' – pages 7.		
Main results	16	(a) Give unadjusted estimates and, if applicable, confounder-adjusted estimates and their precision (e.g., 95% confidence interval). Make clear which confounders were adjusted for and why they were included<br>(b) Report category boundaries when continuous variables were categorized<br>(c) If relevant, consider translating estimates of relative risk into absolute risk for a meaningful time period	Results, subsection 'main results' – pages 7.		
Other analyses	17	Report other analyses done—e.g., analyses of subgroups and interactions, and sensitivity analyses	Results, subsection 'sensitivity analyses' – pages 7 & 8.		
**Discussion**					
Key results	18	Summarise key results with reference to study objectives	Discussion, subsection 'summary of findings' – page 8 & 9.		
Limitations	19	Discuss limitations of the study, taking into account sources of potential bias or imprecision.	Discussion – pages 9 & 10.	RECORD 19.1: Discuss the implications of using data that were not created or collected to answer the specific research question(s). Include	Discussion – pages 9 & 10.

For peer review only

		Discuss both direction and magnitude of any potential bias		discussion of misclassification bias, unmeasured confounding, missing data, and changing eligibility over time, as they pertain to the study being reported.	
Interpretation	20	Give a cautious overall interpretation of results considering objectives, limitations, multiplicity of analyses, results from similar studies, and other relevant evidence	Discussion, pages 8-10.		
Generalisability	21	Discuss the generalisability (external validity) of the study results			
**Other Information**					
Funding	22	Give the source of funding and the role of the funders for the present study and, if applicable, for the original study on which the present article is based	Declarations, page 12.		
Accessibility of protocol, raw data, and programming code		..		RECORD 22.1: Authors should provide information on how to access any supplemental information such as the study protocol, raw data, or programming code.	

*Reference: Benchimol EI, Smeeth L, Guttmann A, Harron K, Moher D, Petersen I, Sørensen HT, von Elm E, Langan SM, the RECORD Working Committee. The REporting of studies Conducted using Observational Routinely-collected health Data (RECORD) Statement. *PLoS Medicine* 2015; in press.

*Checklist is protected under Creative Commons Attribution (CC BY) license.

For peer review only
